# Composition and Sources of Organic Aerosol in Two Megacities in Western China Using Complementary Mass Spectrometric and Statistical Techniques

**DOI:** 10.1021/acsestair.4c00051

**Published:** 2024-07-16

**Authors:** Tianqu Cui, Manousos I. Manousakas, Qiyuan Wang, Gaëlle Uzu, Yufang Hao, Peeyush Khare, Lu Qi, Yang Chen, Yuemei Han, Jay G. Slowik, Jean-Luc Jaffrezo, Junji Cao, André S. H. Prévôt, Kaspar R. Daellenbach

**Affiliations:** † PSI Center for Energy and Environmental Sciences, Paul Scherrer Institute, 5232 Villigen-PSI, Switzerland; ‡ Key Laboratory of Aerosol Chemistry and Physics, Institute of Earth Environment, Chinese Academy of Sciences, Xi’an 710061, China; § Institut des Géosciences de l’Environnement, CNRS, UGA, IRD, Grenoble INP, INRAE, Grenoble 38000, France; ∥ Research Center for Atmospheric Environment, Chongqing Institute of Green and Intelligent Technology, Chinese Academy of Sciences, Chongqing 400714, China

**Keywords:** source apportionment, offline analysis, aerosol mass spectrometer, extractive electrospray ionization, positive matrix factorization, dust

## Abstract

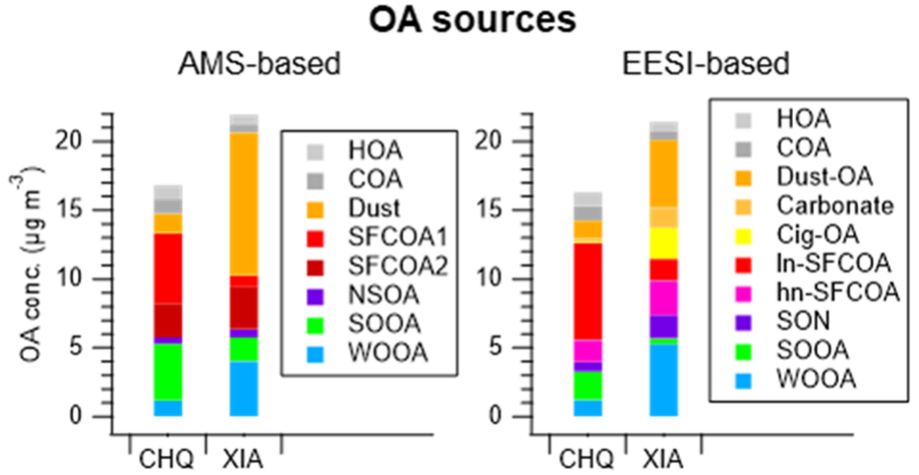

Over 300 daily PM_2.5_ filter samples were collected in two western Chinese megacities, Xi’an and Chongqing, from October 2019 to May 2020. Their aqueous extracts were nebulized simultaneously to an aerosol mass spectrometer (AMS) and a recently developed extractive electrospray ionization (EESI) mass spectrometer, for bulk and near-molecular organic aerosol (OA) composition, respectively. Carbonate was quantified using EESI and a total organic carbon analyzer to separate inorganic carbon from dust. Via isotopically-labelled internal standards and positive matrix factorization, seven water-soluble sources were quantified separately using the AMS- and EESI-based analyses, with consistent types, concentrations, and correlations. These include dust, solid fuel combustion (SFC)-related, nitrogen- (and sulfur-) containing, summer/winter oxygenated OAs, and a cigarette-related OA only in EESI. When accounting for water-solubility, SFC-related OAs were the largest (53%) sources in Chongqing, while dust (consisting of 77% OA and 23% carbonates) was the largest (30%) source in Xi’an. Overall, this study presents one of the first times that complementary mass spectrometric techniques independently resolved consistent OA sources—with added chemical information—over multiple seasons and locations of complex pollution. The methods and quantified sources are essential for subsequent chemical, modelling, and health studies, and policy making for air pollution mitigation.

## Abstract 2

This study used complementary state-of-the-art mass spectrometric and statistical techniques to characterize bulk and near-molecular organic aerosol composition in two western Chinese megacities, resolving consistent source types and concentrations.

## 1 Introduction

Understanding the chemical composition and sources of complex atmospheric organic aerosol (OA) is essential to subsequent climate and health studies and pollution mitigation, but it is largely restricted by the limitations of current analytical techniques. Over the last decade, OA source apportionment was typically based on measurements by the Aerodyne aerosol mass spectrometer (AMS),^1,2^ or aerosol chemical speciation monitor (ACSM),^3^ and in combination with statistical methods such as positive matrix factorization (PMF).^4−6^ It has greatly advanced the quantification of various primary organic aerosol (POA) sources and total secondary organic aerosol (SOA) mass, although resolving individual SOA origins and processes are not really achieved.

A recently developed extractive electrospray ionization time-of-flight mass spectrometer (EESI-TOF) can obtain *in situ* molecular OA fraction with good linear response, minimal thermal decomposition and minimum ionization-induced fragmentation.^7^ The combination of AMS and EESI-TOF has been implemented not only in the field, improving source separation and interpretability,^8−11^ but also adapted to filter-based offline analyses in the laboratory.^12,13^ The offline AMS and EESI simultaneously measure water-soluble organic fraction of ambient particles collected onto conventional particulate matter (PM) quartz filters, enabling the measurement of samples collected at multiple locations. Unlike the traditional offline techniques such as gas- or liquid-chromatography coupled to mass spectrometry (GC-MS or LC-MS) that usually target only several chemical classes as a small fraction of total OA, the AMS can measure major OA through hard ionization, while EESI can provide intact near-molecular information to resolve detailed OA sources. However, since the changing sensitivity during measurement makes quantification challenging, proper corrections shall be performed.

It is of great urgency to understand the pollution sources in highly populated and polluted urban areas. Here, we focus on two megacities, Xi’an and Chongqing, in Western China, other than those more frequently studied megacities in eastern China such as Beijing, Shanghai, and Guangzhou.^14^ In previous source apportionment studies for Xi’an, Huang et al. (2014) used offline AMS and found that haze event was primarily driven by secondary aerosol formation, while the primary aerosol was mainly attributed to dust-related and solid fuel combustion sources.^15^ They resolved the first dust spectral profile of water-soluble constituents with highly oxidized fragments such as C_3_H_7_O_2_^+^ and C_4_H_9_O_2_^+^ proposed as humic species. Elser et al. (2016) applied multi-linear engine (ME-2) and found a large increase of oxygenated organic aerosols (OOA) during the haze events, while biomass burning was the largest OA source.^16^ The aqueous phase processing significantly enhanced sulfate formation but was not equally important for the formation of OOA. Xu et al. (2016) reported a large contribution of dust, not only transported from the nearby Chinese Loess Plateau, but also affected by several primary anthropogenic sources.^17^ Despite the above-mentioned advances in the available instrumentation, detailed composition, origins, and processes of dust and SOA remained insufficiently understood due to a lack of molecular-level mass spectrometric techniques.

In this study, over 300 daily 24-hour PM_2.5_ filters were collected from October 2019 to May 2020, which includes the initial lockdown period due to the Coronavirus disease (COVID-19) and the transition from the winter heating season to warmer weather. It is one of the first times that the OA source apportionment is performed using complementary mass spectrometric techniques, especially over multiple seasons and megacities in China, and interpreted and quantified using a large suite of collocated offline and online measurements.

## 2 Materials and Methods

### 2.1 PM_2.5_ Sample Collection and Gravimetric Analysis

Daily PM_2.5_ samples were collected in Chongqing from January 18 to May 17, 2020 (except March 8, 9, 27), and in Xi’an from October 16, 2019 to May 31, 2020 (except December 18–21; December 24–January 12; March 2, 19, 29–31; April 7, 28–29), for nearly 24 h (approximately from 10:00 A.M. to 9:30 A.M. next day).

In Chongqing, samples were collected in Chongqing Institute of Green and Intelligent Technology, Chinese Academy of Sciences (106.55°E, 29.28°N) as an urban background site. In Xi’an, samples were collected on rooftop of a two-storied building (∼10 m above the ground) on the campus of the Institute of Earth Environment, Chinese Academy of Sciences (108.89°E, 34.23°N), as an urban site influenced by commercial, light industrial, and residential activities.

Overall, a total of 118 Chongqing and 197 Xi’an samples were collected onto quartz-fiber filters (8 inch × 10 inch, QM/A; GE Healthcare, USA) by a high-volume air sampler (Tisch Environmental Inc., USA) at a flow rate of 1.13 m^3^ min^–1^. Three field blank (FB) filters per site were prepared the same way as the exposed PM_2.5_ samples. Upon collection, the filters were measured gravimetrically for mass concentrations with an electronic microbalance (sensitivity: ±1 μg, Sartorius ME 5-F, Gottingen, Germany). They were weighed before and after sampling after equilibration at 20–23 °C and RH of 35%–45%. The exposed samples and field blanks were air-tightly sealed and stored at −20 °C until subsequent chemical analyses.

### 2.2 Instrumentation and Chemical Analyses

Table S1 in the Supporting Information (SI) lists all the offline and online techniques, measurements, and apportioned PM sources reported in this study.

#### 2.2.1 Offline AMS, EESI

The details of the offline AMS and EESI-based measurements and techniques can be found elsewhere.^13,18−25^ Briefly, a 1.13 cm^2^ filter punch area was used for offline mass spectrometric and water-soluble organic carbon (WSOC) measurements. This punch size is sufficient to re-generate aerosol mass concentrations in ambience-relevant levels and achieve enhanced signal-to-noise (S/N) ratios. The filter punches were sonicated in 10 mL ultra-pure water (Milli-Q, 18.2 MΩ cm at 25 °C, total organic carbon <5 ppb, Merck) at 30 °C for 20 min and then vortexed for 60 seconds to optimize extraction efficiency and to achieve homogeneity. The aqueous extracts were filtered using nylon syringe filters (0.45 μm pore size, 13 mm diameter Yeti HPLC filters, Infochroma AG) to remove insoluble materials (including soot particles and filter fibers) that may otherwise clog the experimental setup. The filtered extracts were spiked with 0.15 mL of 200 μg mL^–1^ isotopically-labelled ammonium sulfate (NH_4_)_2_^34^SO_4_ (98%, Aldrich) and ammonium nitrate NH_4_^15^NO_3_ (98.3%, Numelec) to (1) re-generate large enough particle size for effective transmission through the AMS aerodynamic lens; (2) monitor instrument performance and stability during measurements; and (3) serve as internal standards for quantification. The spiked extracts were aerosolized via a nebulizer (APEX-Q, Elemental Scientific Inc.) using synthetic air (PanGas, Switzerland) at a flow rate of 0.7 L min^–1^ regulated by a mass flow controller (Red-y, Vögtlin Instruments GmbH, Switzerland). Every extract was nebulized for 8 min, followed by 12 min of Milli-Q water spray between samples to maintain a clean measurement background between samples. The aerosol was dried via an inbuilt dryer within the nebulizer and further downstream using a Nafion dryer (Perma Pure).

Subsequently, the aerosol stream was split between an Aerodyne high-resolution time-of-flight AMS (HR-TOF-AMS) and an EESI coupled with a long TOF (EESI-LTOF). The AMS was fitted with a PM_2.5_ aerodynamic lens and sampled at 0.07 L min^–1^ in V-mode, with mass resolution of ∼2000 at mass-to-charge ratio (*m*/*z*) 120, to quantitatively measure the bulk non-refractory particle composition (e.g. organics, sulfate, nitrate, ammonium, and chloride). The operating principles, calibration procedures, and analysis protocols of HR-TOF-AMS are described in detail elsewhere.^1,26^ The operation of EESI-TOF has been introduced by Lopez-Hilfiker et al. (2019), and applied to offline analysis by Qi et al. (2020) and lately by Casotto et al. (2022).^7,12,13^ The aerosol sample flow to EESI-LTOF was diluted by a factor of 10 using pure synthetic air to reduce primary ion depletion for better linear response and passed through a charcoal denuder to remove gas-phase species prior to measurement.

#### 2.2.2 Additional Offline Measurements

Organic carbon (OC) and elemental carbon (EC) were measured using 0.526 cm^2^ filter punches via a Thermal/Optical Carbon Analyzer (Desert Research Institute Model 2001, Atmoslytic Inc., Calabasas, CA, USA) with Interagency Monitoring of Protected Visual Environment (IMPROVE_A) thermal/optical reflectance (TOR) protocol.^27,28^ Furthermore, a fraction of the water extracts (3 mL) were used to measure WSOC and water-soluble inorganic carbon (WSIC) (e.g., carbonates and bicarbonates) using the total organic carbon (TOC) analyzer (TOC-L_CPH_, Shimadzu). The organic and inorganic carbon constituents were oxidized to CO_2_ using hydrochloric acid and phosphoric acid, respectively, and measured using a “680 °C combustion catalytic oxidation with nondispersive infrared (NDIR) detection” method. WSOC and WSIC were then used to quantify water-extracted carbon in each sample.

Anhydro-sugars (levoglucosan, mannosan, and galactosan), saccharides (arabitol, glucose, mannitol, sorbitol) were measured using high-performance liquid chromatography with pulsed amperometric detection (HPLC-PAD, Thermo-Fisher ICS 5000+ HPLC, 4 mm-diameter Metrosep Carb 2 × 150 mm column and a 50 mm pre-column). The HPLC-PAD runs were isocratic with 15% of an eluent of sodium hydroxide (200 mM) and sodium acetate (4 mM) and 85% water, at 1 mL min^–1^. This method notably allows the quantification of anhydrous saccharides (levoglucosan, galactosan and mannosan), polyols (arabitol and mannitol), and glucose as tracers of biomass burning and primary biogenic aerosols.^29,30^

Twenty organic acids, including adipic, azelaic, glutaric, glycolic, hydroxylbenzoic, malic, malonic, methylglutaric, 4-methylphtalic, malonic, malic, oxalic, pinic, pinonic, phthalic, pyruvic, succinic, tartaric, vanillic acids, and 3-methyl-1,2,3-butanetricarboxylic acid (3-MBTCA), were also measured via dual ion chromatography coupled to mass spectrometer (IC-MS) ThermoFisher device: 2 INTEGRION + ISQ EC MS detection on the anion line for organic acids, in the negative ion polarity mode (–2700 V) in SIM mode tuned for each species. Limit of detection is lower than 0.1 ng m^–3^ for organic acids in our conditions. Procedural and analytical error is lower than 10%.

Water-soluble anions (NO_3_^–^, SO_4_^2–^, Cl^–^, F^–^) and cations (NH_4_^+^, Na^+^, Mg^2+^, K^+^, Ca^2+^) in sample extracts were measured via ion chromatography (IC, Dionex-600, Dionex, Sunnyvale, CA, USA) equipped with an AS11-HC anion column and a CS12A cation column for separation,^31,32^ or with an ICS-3000 dual-channel chromatograph (Thermo-Fisher) with same columns.^33^ The two sets of IC measurements were highly consistent with Pearson correlation coefficient (*r*) > 0.96 and slope of 1.00–1.07 for NO_3_^–^, SO_4_^2–^, Cl^–^, and K^+^.

The elemental concentrations of As, Ba, Ca, Co, Cr, Cu, Fe, Ga, K, Mn, Ni, Pb, Sc, Se, Sr, Ti, V, and Zn were determined via energy dispersive x-ray fluorescence (ED-XRF) spectrometry (the PANalytical Epsilon 5 ED-XRF analyzer, PANalytical, the Netherlands) and also via inductively coupled plasma triple quadrupole mass spectroscopy (ICP-MS, ThermoFisher Scientific iCAP TQ ICPMS, with ESI SC4DX automatic sampler and FAST valve introduction) following acid digestion. Both ED-XRF and ICP-MS showed consistent results (r > 0.97 for ten elements, > 0.85 for all elements except for Cr and Sc, and slopes of 0.55–1.03 except for Co, Cr, and Sc).

#### 2.2.3 Collocated Online Measurements

The filter-based measurements were complimented with online measurements using two ACSMs: a quadrupole ACSM (Q-ACSM) in Xi’an,^3^ and a TOF-ACSM in Chongqing.^34^ In both instruments, aerosol is sampled through a 100-μm critical orifice, into an aerodynamic lens that focuses the aerosol beam. The non-refractory fraction of the beam is flash vaporized in a standard tungsten vaporizer at ∼600 °C. The vaporized species are ionized by an electron impact (EI) ionization source maintained at 70 eV and analyzed by the Q- or TOF-based mass spectrometer.

A number of particulate elements were measured in 1-h sampling intervals with an Xact 625 ambient metals monitor.^35^ Briefly, the air is sucked through a filter tape with a flow rate of 16.7 L min^–1^. Subsequently the tape is transferred to the analysis section, where it is excited using an x-ray source (Rhodium anode, 50 kV, 50 Watt) in three successive energy conditions that target three different suites of elements. The resulting x-ray fluorescence is measured with a silicon drift detector, and the spectra are analyzed using a proprietary spectral analysis package that takes into account all peaks associated with a given element. During the analysis time, the next sample is collected; finally, the cycle is repeated.

### 2.3 Source Apportionment Analyses

#### 2.3.1 Offline AMS Data

The data analysis steps for offline AMS measurement has been described in detail by Casotto et al. (2022, 2023) and Daellenbach et al. (2016, 2017).^13,20,21,24^ Briefly, raw AMS data was processed in TOF-AMS analysis toolkits: SQUIRREL (SeQUential Igor data RetRiEvaL v. 1.64; D. Sueper, University of Colorado, Boulder, CO, USA) and PIKA (Peak Integration and Key Analysis v. 1.24), to acquire mass spectra of 454 ions over *m*/*z* 12–120. The characteristic mass spectra for each sample was obtained by averaging spectra from the 8 min sampling period and subtracting averaged spectra from the 12 min water background. The error associated with each ion signal involved the standard deviation of the averaging, the minimum error from PIKA accounting for TOF duty cycle correction, and propagated from the water blank subtraction.

The CO_2_^+^ and related peaks (e.g., CO^+^ = CO_2_^+^) were corrected for the nitrate-induced artifacts.^20,36^ To convert to ambient concentrations from offline AMS measurements, previous studies rescaled the averaged organic spectrum of each sample to the quantity of water-soluble OA (WSOA), since the hard electron ionization offers rather balanced response to the different OA constituents.^13,18−24^ Additionally, owing to the non-negligible contribution of carbonates from dust detected as organics by AMS,^18^ this time the AMS spectra were rescaled to
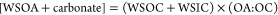
where [WSOA + carbonate] is the water-soluble fraction of OA and carbonate measured by the offline AMS (μg m^–3^) as one entity; WSOC is directly measured using the Shimadzu TOC analyzer (μg m^–3^); WSIC (μg m^–3^) is approximated using carbonate-related ions (e.g., Na_2_HCO_3_^+^ and Na_3_CO_3_^+^) measured by EESI and validated by selected measurements using the Shimadzu TOC analyzer (SI); and (OA:OC) is the OA-to-OC ratio of each spectrum calculated using APES (Analytical Procedure for Elemental Separation) toolkit.^37^ Rescaling AMS OA spectra to [WSOA + carbonate] is considered more reliable than quantifying AMS OA using the internal standards, since the latter may introduce large uncertainty when applying empirical RIEs of OA and inorganics.^13,38^ The mass spectrum of averaged field blanks (three from each city) was subtracted for samples from each city, and the error (standard deviation) was propagated to final PMF input.

Because of ineffective PM_2.5_ collection or offline AMS measurement, five (out of 197) Xi’an samples were excluded for subsequent source apportionment analysis. Sensitivity tests for ions with low S/N ratio (e.g., mean of data-to-error ratio <3) were performed, but the resultant PMF solutions showed negligible difference. Therefore, none of the 454 ions were excluded from the final PMF inputs.

#### 2.3.2 Offline EESI Data

The raw high-time-resolution EESI data was initially time-averaged (20 seconds per spectrum) and processed in Tofware (v3.2.0, TOFWERK AG, Thun, Switzerland) to acquire mass spectra of 2041 ions over *m*/*z* 107–360. For EESI data, the mass spectra exported from Tofware were normalized by intensity of a primary ion adduct (Na_2_I^+^) to correct for changes in sensitivity over time (similar to air beam correction for AMS) and ion suppression issue observed in H_2_O working solutions, before the water blank subtraction. The averaging and error estimation of filter samples and flushing water blanks were performed similarly as for AMS data. Then, the mass spectra of sample and field blank filters were corrected by the intensity of an internal standard adduct (Na_3_[^34^S]O_4_^+^) to correct for variations in nebulizer spray performance over varied concentrations of sample extracts. The data matrix of sample and field blank filters was normalized (or semi-quantified) as
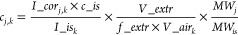
where *c*_*j*,*k*_ is the semi-quantified mass concentration (μg m^–3^) of ion *j* on filter *k*, temporarily assuming response factor of all ions equals to that of the internal standard ion Na_3_[^34^S]O_4_^+^; *I*_*cor*_*j*,*k*_ is the intensity of ion *j* on filter *k* normalized by intensity of an EESI primary ion (Na_2_I^+^); *c*_*is* = 2.1927 μg mL^–1^ is the concentration of isotopically-labeled sulfate spiked as internal standards; *I*_*is*_*k*_ is ion intensity of Na_3_[^34^S]O_4_^+^ on filter *k* normalized by Na_2_I^+^; *V*_*extr* = 10.00 mL of Milli-Q water used for extraction; *f*_*extr* = 0.002729 is the fraction of extracted filter area (1.13 cm^2^) to the entire exposed filter area (414 cm^2^); *V*_*air*_*k*_ is the sampled air volume (m^3^) of filter *k*; *MW*_*j*_ is the effective molecular mass (after excluding Na^+^, H^+^, NaI, or H_2_O adducts or clusters) of ion *j*; and *MW_is_* = 97.9475 is the monoisotopic molecular mass of [^34^S]O_4_. The last term [*MW_j_*/*MW_is_*] serves as correction factor from intensity (or ion counts) to mass that is relative to *MW_is_*. The error matrix of PMF inputs was calculated using σ_*j*,*k*_ (error of ion *j* on filter *k*) in the same way. With such corrections and subsequent semi-quantification steps, the sulfate quantified by EESI agrees strongly with that measured by IC (slope = 1.03, improved from *r* = 0.84 to *r* = 0.99, Figure S1), proving the effectiveness of utilizing internal standards in (semi-)quantification of EESI data. Additionally, remarkable agreements were achieved for both sulfate and nitrate quantifications using EESI (and AMS) vs. IC, with slopes from 0.97–1.07, *r* > 0.98 (Figure S1).

Throughout PMF solution optimization, a set of criteria was developed to filter out ions with relatively low S/N or sample-to-blank ratio (e.g., lowest 25% in each criterion, SI). As a result, 1127 ions were retained in the PMF inputs, out of the 2041 ions originally fitted in Tofware.

#### 2.3.3 Water-Soluble Carbonate Carbon in Aerosol Samples

Atmospheric dust consists of crustal (e.g., Si, Al, Fe, Ca) and other metallic elements, carbonates, as well as organic constituents. Although dust primarily contributes to coarse PM, source apportionment of offline filter measurements revealed a dust factor among major sources of PM_2.5_ in Xi’an, contributing 46.3% of PM mass in January 2013,^15^ and 19.4% in January and February 2010.^17^ The high dust contributions were attributed to dust storms originating from deserts in northwest China, and fugitive dust from construction sites and unpaved roads. A dust factor was also identified using offline AMS for PM_10_ in Estonia,^18^ where the carbon in the dust factor was all treated as inorganic carbonates and thus retracted from WSOC. Similar to Xi’an, a preliminary dust factor was also identified in PM_2.5_ in Marseille using offline AMS but excluded inorganic carbon from WSOC.^23^

In this study, it is vital to quantify carbonate-related carbon in Xi’an that is strongly affected by dust, so that carbonates could be distinguished from OA, in estimating PM source contributions. Table S2 lists carbonate carbon and OC (or OA) to be measured separately or as one quantity by the multiple techniques in this study. Here, carbonate carbon in all filters was measured using EESI-LTOF. Four carbonate-related ions were identified: Na_2_HCO_3_^+^, Na_3_CO_3_^+^, Na_2_HCO_3_(NaI)^+^, and Na_3_CO_3_(NaI)^+^. Na_2_HCO_3_^+^ correlates best with WSIC (*r* = 0.96, *n* =18, Figure S2) from selected filters and was determined to quantify carbonates via EESI measurements.

#### 2.3.4 ACSM Data

Source apportionment of the ACSM data mainly assisted in interpreting the offline AMS and EESI factor solutions and to estimate OC from water-insoluble OA (WIOA) sources for mass closure and recoveries. Raw data was analyzed using ACSM local (v1.6.1.3) for the Q-ACSM in Xi’an, and Tofware (v2.5.13) for the TOF-ACSM in Chongqing. The processed data was averaged over 30 min. Relative ionization efficiency (RIE) was experimentally determined for ammonium and sulfate, while for chloride and organics the standard RIE of 1.4 was used. The default collection efficiency (CE) = 0.5 was applied based on previous work.^39^

#### 2.3.5 Positive Matrix Factorization

Positive matrix factorization (PMF) is a bilinear receptor model with non-negativity constraints used to separate the data matrix into different factors of emission sources or atmospheric processes.^40^ The data matrix presents the time series of mass spectra (or a group of PM chemical species) from filter-based or online measurements. In this study, PMF is performed in the multilinear engine (ME-2),^41^ with model configuration and post-analysis implemented in the Source Finder (SoFi, Pro v8, Datalystica Ltd.) toolkit.^42,43^

For the offline AMS and EESI data, the error matrix was weighted using a step function, as proposed by Paatero and Hopke (2003),^44^ and a cell-wise S/N ratio for single elements in the PMF input was calculated.^45^ “Bad” signals with S/N below 0.2 were down-weighted by a factor of 10, “weak” signals with S/N between 0.2 and 1 were down-weighted by a factor of 2, and the CO_2_^+^-related ions in AMS were also down-weighted to avoid over-counting.^6^

To achieve optimized solutions, a number of key steps of source apportionment were performed.^20^ Preliminary PMF runs were performed to determine the number of factors and general types of factors (*Q*/*Q*_exp_ in Figure S3). PMF runs using reference profiles as constraints were performed. For AMS this included hydrocarbon-like OA (HOA)^4^ and coal combustion OA (CCOA) from offline AMS.^46^ However, the final solution was obtained without any constraints. For EESI, a profile identified as summer oxygenated OA (SOOA) from a preliminary 18-factor solution was retrieved and used as a constraint (*a*-value = 0.05) to resolve this optimized SOOA factor from solutions with fewer factors. A profile strongly correlating (*r* = 0.83) with an AMS nitrogen- and sulfur-containing OA (NSOA) from an 11-factor solution was obtained as a constraint (*a*-value = 0.05) for the same purposes. The ion of C_10_H_15_N_2_^+^ and C_10_H_15_N_2_(NaI)^+^ were constrained to be zero in all factors but one, to better resolve one factor related to cigarette smoking.

Once an AMS (or EESI) base-case solution was determined, which is both environmentally reasonable and comparable between the two techniques, criterion-based sensitivity tests were performed to evaluate the uncertainty of the PMF solution via bootstrapping.^20,42^ The selection criteria are set mainly by correlation with the base-case solution and are listed in Tables S3 and S4. Uncertainties of the selected bootstrapped runs for AMS and EESI are shown in Figure S4 and S5, respectively. The final AMS solution was averaged from criterion-based selection of 698 out of 1000 bootstrapped PMF runs, with relative standard deviation (RSD) from 6.6%–13.4%. The final EESI solution was averaged from 259 out of 1000 bootstrapped runs, with RSD from 4.0%–10.9%.

The ACSM data source apportionment analysis was conducted by following the standard protocol established by Chen et al. (2022).^47^ First, PMF pretests were executed to identify the OA sources and retrieve reasonable source profiles. HOA and cooking OA (COA) mass spectra were constrained using the one from Crippa et al. (2013),^4^ while biomass burning OA (BBOA) and CCOA profiles were retrieved by constraining the time series of the factors with *m*/*z* 60 and 115, respectively. Bootstrap resampling strategy with random *a*-values was used to investigate the stability of the solution; 1000 bootstrapped runs were performed and the environmentally reasonable solutions were chosen by criteria-based selection. Finally, the average source profiles resulting from the bootstrapped runs were used to constrain the profiles of the POAs for the subsequent “rolling PMF” runs with an *a*-value = 0.2. Unlike conventional PMF assuming static source profiles, rolling PMF analysis occurs on a smaller time window (e.g., 14 days) to roll over the whole dataset with a certain step, which allows the factors to adapt to temporal changes.^43^ The goodness of the fit was evaluated by examining the diurnal variations of the source contributions and the correlation to external data.

#### 2.3.6 Quantification and Recoveries from WSOA to OA

First, the EESI PMF factors were converted to water-soluble concentrations in μg m^–3^. The EESI spectra as PMF input were not individually scaled to a total quantity (i.e., [WSOA + carbonate] as done for the AMS spectra), because of the chemical incompleteness of electrospray ionization and different sensitivity between species.^7,48^ Instead, time series of the PMF-resolved EESI factors, along with carbonate carbon, were regressed to the time series of [WSOA + carbonate] = (WSOC + WSIC) × (OA:OC), using a multiple linear regression (MLR) model on a Bayesian statistical platform implemented in Python (PyStan). Here, the fitted coefficients reflect the relative sensitivity of the semi-quantified WSOA factors and carbonate (Table S5), leading to good fitting results (slope = 0.97, *r* = 0.98, Figure S6).

Once the final offline AMS WSOA factors were obtained, recoveries (i.e., water-solubility in %) of the factors were assessed using the same MLR model by fitting WSOC to total OC, after accounting for OC from entirely water-insoluble factors (i.e., HOA and COA retrieved from ACSM). As described in SI, OC in HOA and COA was estimated based on f_*m*/*z*=44_ (fraction of *m*/*z* 44 to the profile),^49^ averaged to 24-hour corresponding to filter collection periods, corrected for RIE,^50,51^ and rescaled to OC measured on filters (Figure S7). The resolved EESI WSOA factors and carbonate were analogously regressed to the sum of recovered AMS OA concentrations. For both AMS and EESI recoveries, the fitted coefficients representing factor-specific recovery rates were constrained to be between 1 and 10, corresponding to a 10%–100% water solubility. More details about the MLR models and fitted results are included in the SI.

## 3 Results and Discussion

### 3.1 WSOA Sources from Offline AMS and EESI

**Figure 1:**
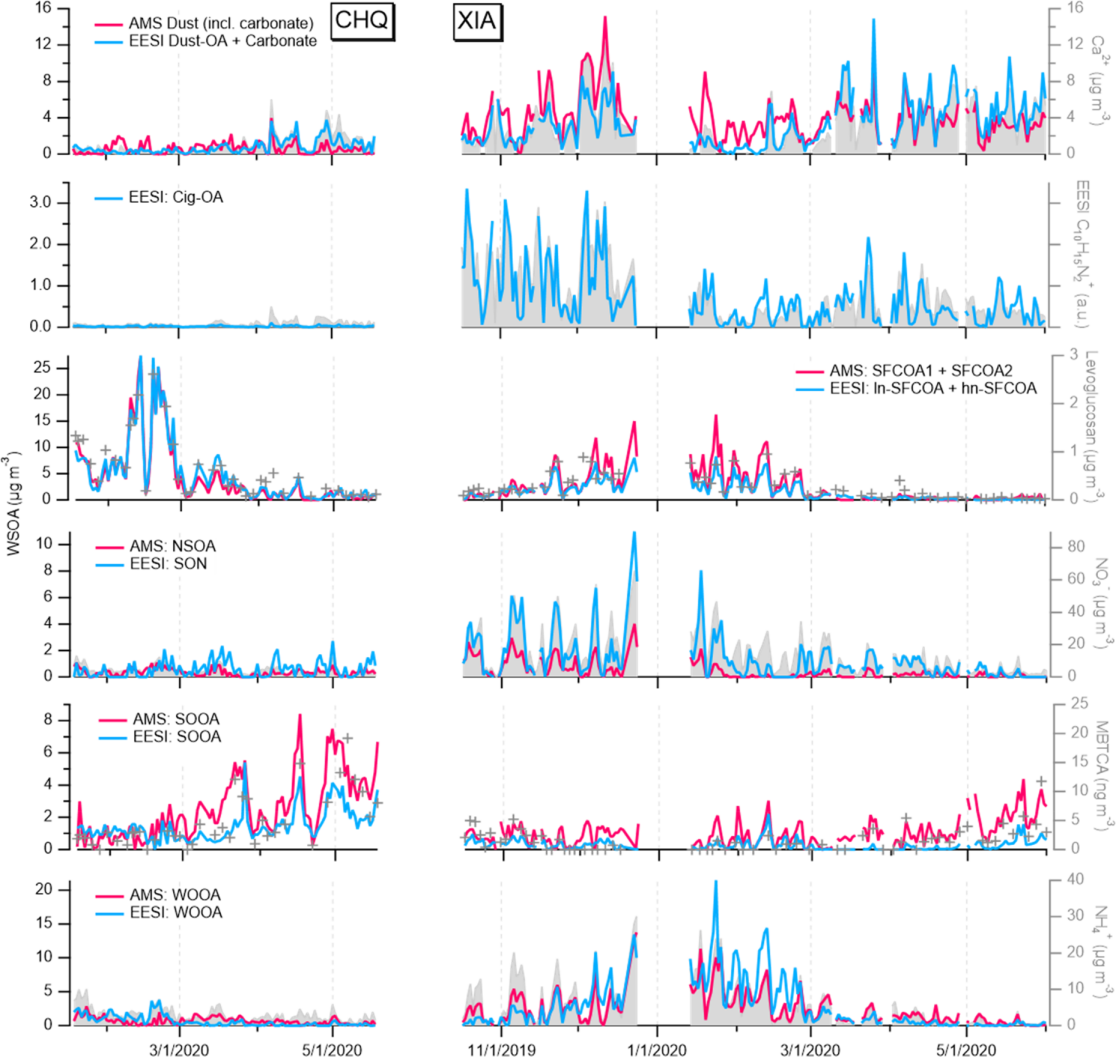
Concentration of WSOA sources between AMS (red) and EESI (blue) in Chongqing (CHQ, left) and Xi’an (XIA, right). A tracer corresponding to the source is appended in each panel (grey, right *y*-axis).

**Figure 2:**
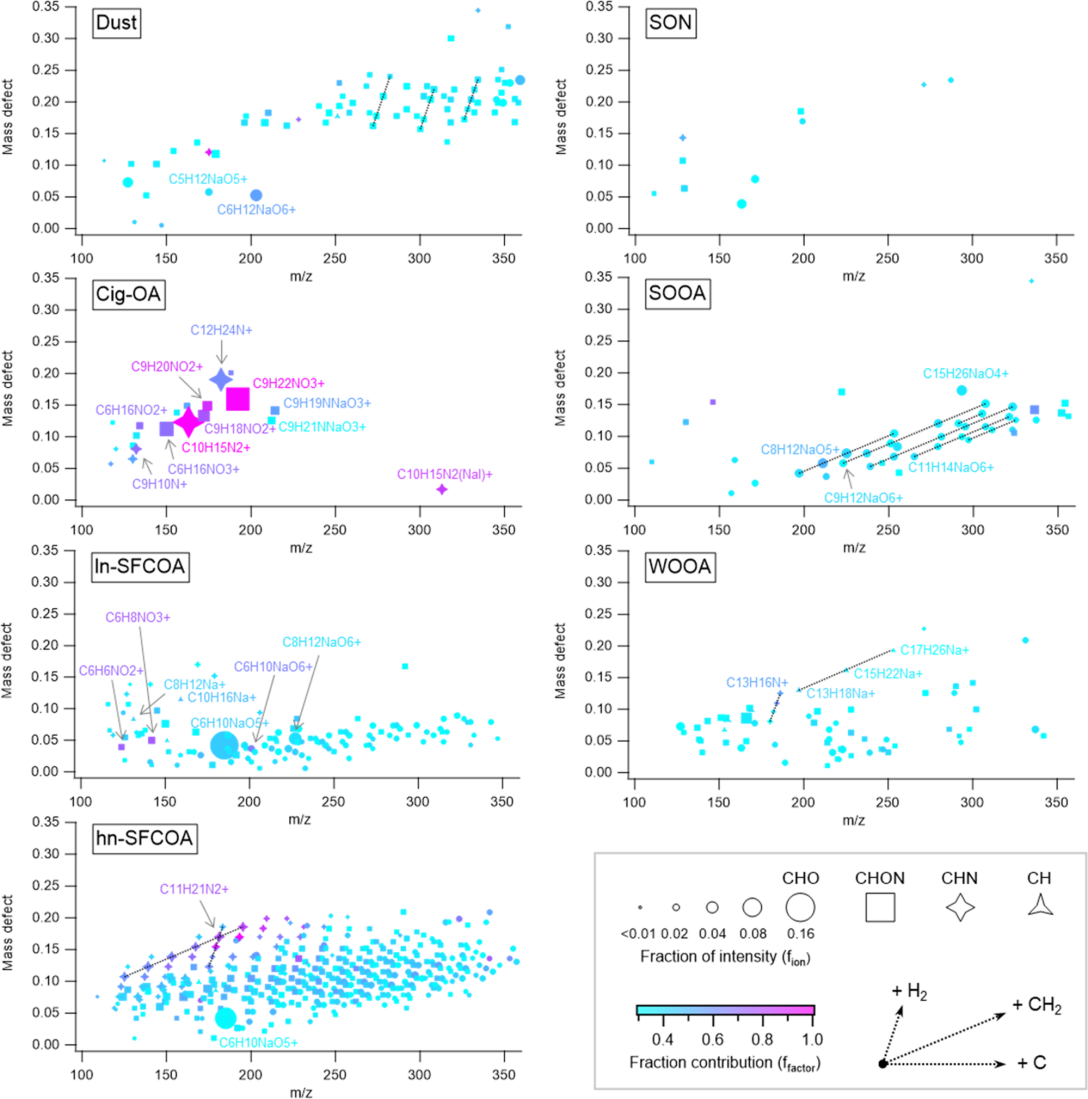
Mass defect of selected fingerprint ions in each EESI factor profile. Marker color shows fraction contribution of the factor (f_factor_) to each ion. Ions with f_factor_ < 0.3 are not displayed. Marker size indicates fraction contribution of the ion (f_ion_) to the factor profile. Marker shape represents elemental composition of the ion: CHO, CHON, CHN, or CH.

The AMS-based analysis revealed six water-soluble sources contributing to OA in PM_2.5_ in both Xi’an and Chongqing, including a dust factor, two solid fuel combustion-related OAs (SFCOAs), a nitrogen- and sulfur-containing OA (NSOA), a summer oxygenated OA (SOOA), and a winter oxygenated OA (WOOA). The EESI-based analysis also identified these sources and an additional cigarette OA in a 7-factor solution. The PMF solutions were finalized using the methods stated in Section 2.3.2. Figures 1–2 show the time trends and molecular-level chemical profiles of the identified sources. Note that these water-soluble sources consisted of WSOA and carbonates, with the unit of μg m^–3^. Factors and components from the same origins have been merged for comparison between AMS and EESI. For example, AMS Dust contained carbonate and thus should be compared with EESI Dust-OA plus carbonate. Time series and profiles of individual factors as direct PMF outputs are shown in Figure S8 and S9 for AMS, and in Figure S10 and S11 for EESI.

#### 3.1.1 Dust

Dust was observed as a major contributor in Xi’an via both offline AMS and EESI-based measurements. While the AMS dust factor consisted of OA and carbonate detected as CO_2_^+^-related fragments (Table S2),^18^ the EESI Dust-OA contained only organic constituents, since carbonate was quantified separately as described in Section 2.3.2. The low dust concentrations in Chongqing was likely due to limited outreach of dust storms from the northwest deserts. Such difference between Xi’an and Chongqing is consistent with prior studies.^15,17^

The AMS dust profile was dominated by CO_2_^+^ and its related ions (i.e., O^+^, OH^+^, H_2_O^+^, CO^+^, Figure S9), and characterized by the highest OA:OC ratio = 2.07, likely because carbonate and bicarbonate were detected primarily as CO_2_^+^ through thermal decomposition and/or electron ionization in AMS.^18,23^ In the EESI Dust-OA profile (Figure 2, S11), C_5_H_12_O_5_Na^+^ and C_6_H_12_O_6_Na^+^ were largely explained by this factor, with high fraction contribution of this factor to the ion (f_factor_ = 0.39 and 0.56, respectively), are tentatively identified as arabitol and glucose in plant debris from primary biogenic origins,^30^ and were possibly re-suspended with transported or fugitive dust. Yet, the correlations with measured arabitol and glucose are weak (*r* = 0.38 and 0.16, respectively) implying that the concentrations of the latter are not fully explained by dust factor.

The concentrations of water-soluble dust are consistent between AMS and EESI (slope = 0.78, *r* = 0.68, Figure S12), indicating that the two techniques can independently resolve the same source. This correlation is stronger than that of AMS Dust either with EESI Dust-OA (*r* = 0.61, Figure S13) or with carbonate (*r* = 0.60), suggesting the importance of separating organic and carbonate constituents in dust. The AMS and EESI dust factors correlate strongly with water-soluble calcium (*r* = 0.78 and 0.75, respectively, Figure S12), and with other mineral or road dust elements, such as total Ca (*r* = 0.77 and 0.75, Figure S13), Fe (*r* = 0.76 and 0.78), Mn (*r* = 0.80 and 0.74) and Ti (*r* = 0.71 and 0.79), indicating a large contribution of their mineral and fugitive origins.

#### 3.1.2 Cigarette OA

A cigarette OA factor (Cig-OA) was resolved only by EESI, though a few cigarette-related fragment ions were resolved in the AMS NSOA factor discussed later. The Cig-OA factor was mainly observed in Xi’an and barely in Chongqing (Figure 1, S10), likely because the Xi’an site was near city center with intense human activities, while the Chongqing site was suburban. The concentration was suddenly reduced since January 2020, probably due to the initial COVID-19 lockdown and restrictions on human activities.

The EESI profile is dominated by C_10_H_15_N_2_^+^ since it was constrained to appear only in this factor, resulting in fraction contribution of this ion intensity to the factor (f_ion_) = 0.198 (Figure 2, S11). This ion was identified as protonated nicotine from cigarette smoking in previous offline and online studies using EESI-TOF.^9,11,12^ It is worth mentioning that one compound can be ionized either with Na^+^ or H^+^ (e.g., C_9_H_22_NO_3_^+^ and C_9_H_21_NO_3_Na^+^), and with an additional NaI cluster such as C_10_H_15_N_2_(NaI)^+^. C_6_H_10_O_5_Na^+^ was previously found in Cig-OA, which was mostly identified as a BBOA marker species,^8−12^ but with low f_factor_ = 0.05 here, indicating that this factor barely explained the variation of C_6_H_10_O_5_Na^+^. Other formulae of major ions, in decreasing order of f_ion_, included: C_9_H_22_NO_3_^+^ (f_factor_ = 0.98, Figure 2), C_6_H_16_NO_3_^+^ (f_factor_ = 0.69), C_9_H_18_NO_2_^+^ (f_factor_ = 0.77), C_12_H_24_N^+^ (f_factor_ = 0.62), and C_9_H_20_NO_2_^+^ (f_factor_ = 0.86). C_9_H_22_NO_3_^+^, C_6_H_16_NO_3_^+^, and C_12_H_24_N^+^ can be tentatively proposed as amines, such as triisopropanolamine, triethanolamine, and dicyclohexylamine, respectively, that are commonly added in daily chemical products and emitted from anthropogenic origins along with cigarette consumption.

The time series of Cig-OA correlates strongly with C_10_H_15_N_2_^+^ due to the PMF constraint (*r* = 0.89, Figure S13), followed by Zn (*r* = 0.64), *n*-alkanes (*r* = 0.58), and Mn (*r* = 0.58), suggesting the anthropogenic origins such as cigarette smoking and usage of chemical and industrial products.

#### 3.1.3 Solid Fuel Combustion Related OAs

Both the AMS and EESI resolved two factors related to partially aged emissions from different solid fuel combustion (SFC), namely SFCOA1 and SFCOA2 from the AMS-based analysis, and low-nitrogen SFCOA (ln-SFCOA) and high-nitrogen SFCOA (hn-SFCOA) from the EESI-base analysis. The summed concentration agreed well between the AMS- and EESI-based analyses (slope = 0.89, *r* = 0.95, Figure S3), showing consistent identification of solid fuel sources between the two independent techniques. In sum, it appears likely that the AMS SFCOA1 and the EESI ln-SFCOA are more impacted by biomass burning, while the AMS SFCOA2 and the EESI hn-SFCOA are originated from additional solid fuels such as coal.

Both of the AMS factors have characteristic features of biomass and other SFC-OA marker ions represented by C_2_H_4_O_2_^+^ at *m*/*z* 60 and C_3_H_5_O_2_^+^ at *m*/*z* 73 (Figure S8 and S9),^52^ yet they also show relatively high CO_2_^+^ signal indicating substantial aging. Their OA:OC ratios (1.82 and 1.79 for SFCOA1 and SFCOA2, respectively) exceed previously observed values for fresh biomass burning emissions (1.74).^20^ Their time series shows expected seasonal variation with higher wintertime concentrations and correlates well with the ACSM BBOA factor (r from 0.83–0.84, Figure S13). In addition, SFCOA1 correlates with ACSM BBOA but not with ACSM CCOA, and better than SFCOA2 with levoglucosan (*r* = 0.92 for SFCOA1 vs. *r* = 0.72 for SFCOA2), mannosan (0.89 vs. 0.68), pyruvic acid (0.88 vs. 0.62), and succinic acid (0.82 vs. 0.47). On the other hand, SFCOA2 correlates more strongly with primary emissions of fossil fuel and anthropogenic related factors and tracers than SFCOA1 did, such as with ACSM CCOA (*r* = 0.33 for SFCOA1 vs. *r* = 0.80 for SFCOA2), PAHs (0.01 vs. 0.76), EC (0.35 vs. 0.71), *n*-alkanes (–0.10 vs. 0.42), phthalic acid (0.25 vs. 0.76), methylphthalic acid (0.21 vs. 0.78), vanillic acid (0.64 vs. 0.83), hydroxybenzoic acid (0.51 vs. 0.94), and galactosan (0.73 vs. 0.82). The concentrations and fraction of these three anhydro-sugars can be found in Figure S14. To summarize, SFCOA1 was mainly influenced by partially aged biomass burning-related emissions containing anhydro-sugars and oxygenated low-molecular-weight acids, while alongside biomass burning, SFCOA2 was strongly affected by complex emissions such as coal combustion.^46,53^

The two EESI SFC-related factors also have common and distinctive features. Both profiles contain prominent BBOA marker ions including C_6_H_10_O_5_Na^+^ at *m*/*z* 185, and C_8_H_12_O_6_Na^+^ at *m*/*z* 227 (Figure S11).^8−12^ C_6_H_10_O_5_ includes isomers of anhydrous sugars such as levoglucosan, mannosan, and galactosan from BBOA emissions. C_8_H_12_O_6_ was proposed as a derivative of syringol from wood-burning OA.^54^

The ln-SFCOA profile is driven by ions with relatively low mass defect at around 0.05, while hn-SFCOA consists of more CHN and CHON compounds and larger mass defect from 0.05–0.20 (Figure 2). Specifically, some reduced nitrogen-containing species in hn-SFCOA can be easily ionized in EESI positive ion mode and detected in the protonated form ([C_*x*_H_*y*_N_2_+H]^+^, Figures 2 and S11). This series of nitrogen-containing compounds were tentatively identified as N-heterocyclic alkaloids that are naturally produced by plants and released in biomass burning emissions,^55^ and also found in brushwood and dung burning emissions,^56,57^ rather than secondary organic nitrogen compounds (e.g., imines) formed in aqueous particle phase from dicarbonyls in the presence of ammonium.^58,59^ As for the time series, both EESI factors correlate moderately with the AMS SFCOA2 (*r* = 0.59–0.68, Figure S13), with ln-SFCOA correlating more strongly with the AMS SFCOA1 than hn-SFCOA does (*r* = 0.94 vs. 0.21). ln-SFCOA also correlates more strongly with typical BBOA factor and tracers than hn-SFCOA does, such as with ACSM BBOA (*r* = 0.92), AMS C_2_H_4_O_2_^+^ (*r* = 0.90), levoglucosan (*r* = 0.93), mannosan (*r* = 0.89), also with pyruvic acid (*r* = 0.91) while hn-SFCOA correlates more strongly than ln-SFCOA with ACSM CCOA (*r* = 0.71), vanillic acid (*r* = 0.79), hydroxybenzoic acid (*r* = 0.78), Cl^–^ (*r* = 0.58), F^–^ (*r* = 0.67), and PAHs (*r* = 0.65). Above all, ln-SFCOA is essentially attributed to biomass burning, while hn-SFCOA is strongly impacted by coal combustion emissions though mixed with combustion emissions from nitrogen-containing fuels.

#### 3.1.4 Nitrogen- (and Sulfur-) Containing OAs

The AMS NSOA and EESI secondary organic nitrogen (SON) factors are both nitrogen- (and sulfur-) containing OAs that correlate strongly with each other (*r* = 0.83, Figure S12). The absence of sulfur-containing species in EESI is because organic sulfur compounds have low ionization efficiency in the positive ion mode of electrospray.

The AMS NSOA factor profile is characterized by a low O:C ratio = 0.23, absence of CO_2_^+^ ion signal, presence of C_2_H_4_O_2_^+^, and large contributions from CHN, CHON, and CHOS ion families (Figure S9). The prominent CHOS ions are CH_3_SO_2_^+^ at *m*/*z* 79 and CHSO^+^ at *m*/*z* 61 that have been previously attributed to primary traffic-related sources of coarse PM in urban areas.^12,19,20^ C_5_H_10_N^+^ at *m*/*z* 84 and C_2_H_4_N^+^ at *m*/*z* 42 were initially proposed as nicotine fragments from cigarette-smoking,^60^ but also observed in laboratory-controlled primary coal combustion and certain wood burning.^46^

The AMS NSOA and EESI SON correlate strongly with secondary inorganic species (Figure S13), such as NH_4_^+^ (*r* = 0.69 and 0.80, respectively) and NO_3_^–^ (*r* = 0.74 and 0.87), implying the process of particulate organic nitrogen formation. It is worth noting that the aerosol liquid water content (ALWC) modeled using ISORROPIA II^61^ correlates with these two factors much stronger (*r* = 0.50 and 0.58, respectively with the AMS NSOA and EESI SON) than any other factors, suggesting the formation of organic nitrogen and sulfur might be involved with aqueous processes.

#### 3.1.5 Summer Oxygenated OAs

An SOOA factor is resolved by both AMS and EESI (slope = 0.46, *r* = 0.83, Figure S12). The SOOA factor significantly enhanced during spring, especially in Chongqing, probably because the suburban Chongqing site is surrounded by dense vegetation and is warmer than Xi’an by 5–10 °C in springtime (Figure S14). The AMS SOOA correlates more strongly with 3-MBTCA, temperature, and temperature-approximated biogenic SOA^62^ (*r* = 0.84, 0.69, 0.73, respectively, Figure S13) than the EESI SOOA does (*r* = 0.78, 0.55 and 0.51), suggesting that the SOOAs is likely derived from biogenic precursors such as terpenes and isoprene.

The AMS SOOA profile shows high contributions of C_2_H_3_O^+^ and CO_2_^+^, an OA:OC ratio = 1.86 (Figure S9), and mass spectral fingerprints similar to that of previous offline SOOA spectrum.^12,20^ The EESI SOOA profile shows a clear pattern of C_*x*_H_*y*_O_*z*_ series containing C_7_–C_16_ compounds (Figure 2, S11), also similar to the previous SOOA and biogenic SOA spectra from ambient and laboratory measurements.^9,12,13,63^

The dominant contribution of biogenic sources to SOOA is supported by EESI-based measurements, where the EESI profile shows prominence of C_4_H_6_O_5_ (e.g., malic acid), C_5_H_12_O_4_ (e.g., 2-methyltetrol), C_5_H_8_O_5_ (e.g., 3-hydroxyglutaric acid) that were also observed as major products in southeastern US relating to isoprene- or monoterpene-derived SOA formation.^64,65^ C_7_H_10_O_5_ (e.g., 3-acetylpentanedioic acid),^66^ C_7_H_12_O_5_, C_8_H_12_O_5_, C_9_H_12_O_5_, C_9_H_14_O_5_, C_8_H_12_O_6_ (e.g., 3-MBTCA),^67^ C_10_H_14_O_5_, C_9_H_12_O_6_, and C_10_H_16_O_6_ are consistent with the major compounds related to monoterpene biogenic SOA using online and offline EESI-TOF studies in a remote boreal forest in Finland and in Zurich, Switzerland.^9,11,12,63^ The C_12_–C_15_ formulae, including C_12_H_20_O_5_, C_13_H_20_O_5_, C_15_H_26_O_4_, C_15_H_24_O_5_, C_15_H_24_O_6_, are tentatively proposed as sesquiterpene oxidation products, which were also observed in previous EESI biogenic SOA-related chemical profiles.^12,13^

#### 3.1.6 Winter Oxygenated OAs

A winter-oxygenated OA (WOOA) factor is resolved via both of the AMS- and EESI-based analyses, with elevated concentrations during winter periods (Figure S8 and S10), and of large consistency between the two techniques (slope = 1.15, *r* = 0.81, Figure S12).

Compared to the AMS SOOA, the AMS WOOA profile has a higher OA:OC ratio = 1.95 (Figure S9), lower f_C2H3O+_, and equally large contribution of CO_2_^+^, which is in line with previous results on offline AMS WOOA.^20^ Similar to the EESISFCOAs, the EESI WOOA profile is dominated by C_6_H_10_O_5_Na^+^ and C_8_H_12_O_6_Na^+^ but with lower f_ion_ (Figure S10), suggesting the large contribution from more aged (less primary as BBOAs) solid fuel combustion rather than biomass. The ions with largest f_factor_ in this profile have high H:C and low O:C ratios, such as C_11_H_15_NNa^+^ (f_factor_ = 0.60, Figure 2), C_11_H_17_NNa^+^ (f_factor_ = 0.55), C_13_H_18_Na^+^ (f_factor_ = 0.43), and C_11_H_14_Na^+^ (f_factor_ = 0.40). These ions can be related to PAH derivatives, supported by the correlation with PAHs (*r* = 0.70, Figure S13).

The time series of AMS and EESI WOOAs correlate with secondary inorganic species including NH_4_^+^ (*r* = 0.90 for AMS and *r* = 0.72 for EESI, Figure S13), NO_3_^–^ (*r* = 0.84 and 0.65), and SO_4_^2–^ (*r* = 0.78 and 0.61) measured by IC, as well as with oxidation products of aromatic precursors such as phthalic acid (*r* = 0.68 and 0.77) and methylphthalic acid (*r* = 0.69 and 0.83), further supporting that WOOA represents strongly oxygenated anthropogenic SOA.

### 3.2 Recovered OA Sources

Factor-specific recoveries (i.e., water-solubility) were computed and found remarkably consistent between the offline AMS and EESI (Table S6), and are in line with those computed for the same sources resolved in previous offline AMS studies.^18−21^ Specifically, solubility of AMS Dust = 37.8% vs. EESI mass-weighted OA and carbonate = 53.0%, AMS SFCOA1 = 59.0% vs. EESI ln-SFCOA = 64.2%, AMS NSOA = 79.4% vs. EESI SON = 79.5%, AMS SOOA = 61.0% vs. EESI SOOA = 71.9%, AMS WOOA = 60.0% vs. EESI WOOA = 53.5%. The EESI SON is the most water-soluble factor, likely due to the potential formation via aqueous chemistry. Carbonate is fitted to be nearly all soluble (97.2%).

From the AMS factors, OC mass closure is achieved between recovered and measured OC (slope = 0.94, *r* = 0.95, Figure S15). The mass closure of secondary OC is assessed by fitting recovered OC in the AMS factors (except for Dust) to the ACSM oxygenated OC (slope = 0.96, *r* = 0.94, Figure S15). The AMS Dust constituents, as a mixture of OA and carbonate typically not detected by ACSM due to particle bouncing, is assumed to be fully primary and thus excluded from this fitting. By combining the offline and online analyses, the actual fraction of secondary OC in the AMS SFC-related and “secondary” OA factors has been assessed: 37% OC in SFCOA1 is fitted to be secondary, as well as 42% OC in SFCOA2, while OCs in NSOA, SOOA, and WOOA are almost all secondary (100%, 98%, and 100%, respectively). At last but not least, for EESI, the sum of recovered OA factors and carbonate is consistent with that in AMS (slope = 0.99, *r* = 0.95, Figure S16).

**Figure 3:**
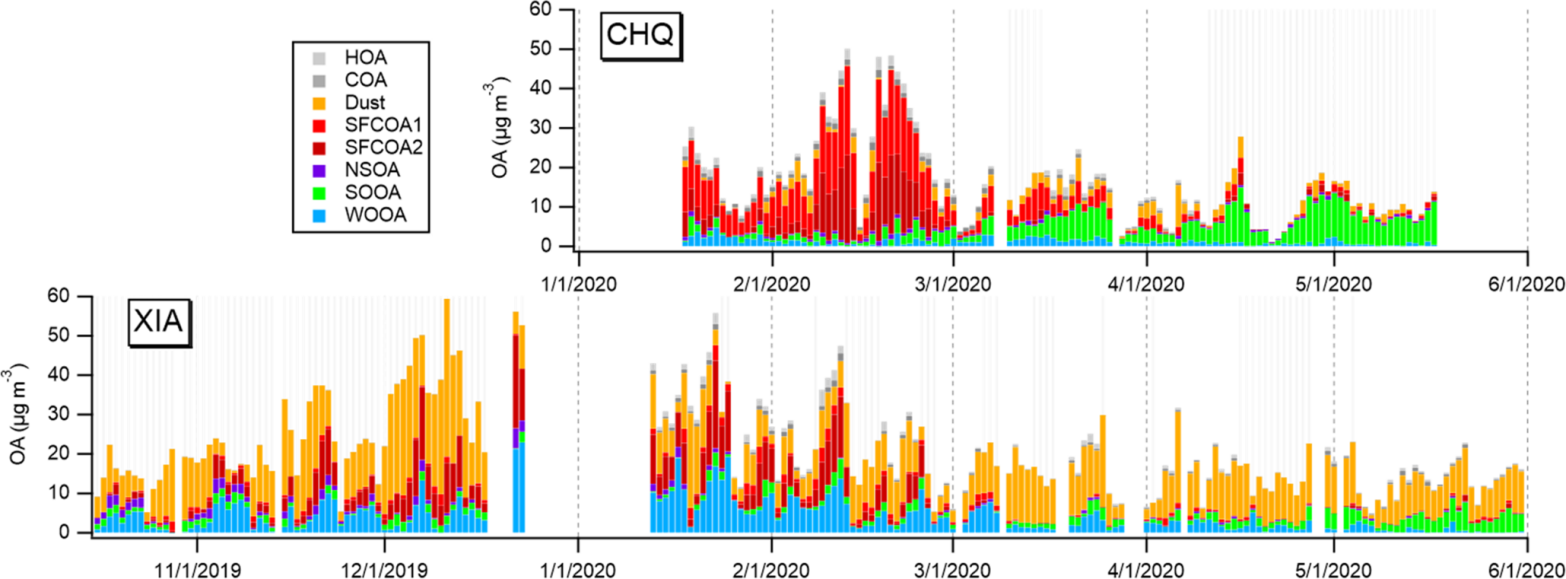
Recovered OA sources in Chongqing (CHQ, top) and Xi’an (XIA, bottom) using AMS-based analysis. AMS Dust contains carbonates. HOA and COA from ACSM data are unavailable in ∼45% of the days as indicated by vertical light grey lines.

These recovery rates were then used to compute OA concentration for each WSOA factor. Time series of AMS OA factors are displayed in Figure 3 (EESI OA factors in Figure S17). Clear seasonal variation in OA sources was visible in both Chongqing and Xi’an. In winter, SFC-related OA factors were the major contributors to OA, while in summer SOOA dominated in Chongqing and dust dominated in Xi’an.

**Figure 4:**
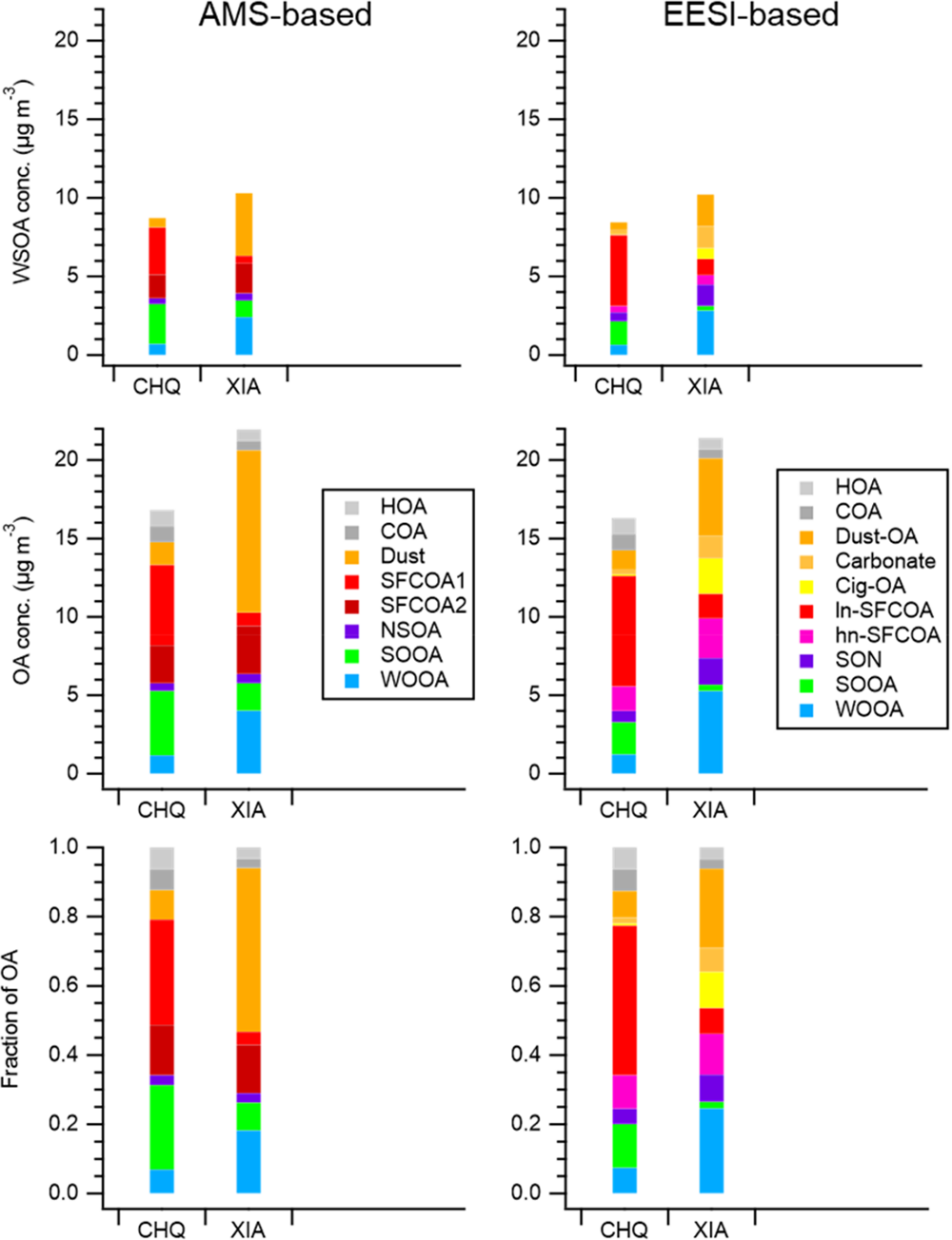
Concentration of WSOA (top) and OA (middle), and fraction of OA sources (bottom) in Chongqing (CHQ) and Xi’an (XIA) using either AMS (left) or EESI (right). The water-insoluble OA sources (i.e., HOA, COA) are retrieved from ACSM source apportionment.

OA concentrations were averaged over the studied periods and shown in Table 1 and Figure 4. The concentration and fraction of OA sources common to both the EESI and AMS data are generally consistent. The additional Cig-OA only resolved by the EESI data was a minor contributor (6.8%) to OA.

**Table 1:** Averaged Concentration (μg m^–3^) and Fraction of WSOA and OA Sources in Chongqing (CHQ) and Xi’an (XIA) Using Either AMS- or EESI-Based Analysis^a^

		WSOA						OA					
		all (*n* = 310)		CHQ (*n* = 118)		XIA (*n* = 192)		all (*n* = 170)		CHQ (*n* = 75)		XIA (*n* = 95)	
		*avg.*	*frac.*	*avg.*	*frac.*	*avg.*	*frac.*	*avg.*	*frac.*	*avg.*	*frac.*	*avg.*	*frac.*
**ACSM**	HOA							0.88	4.4%	1.05	6.3%	0.74	3.4%
	COA							0.77	3.8%	1.01	6.0%	0.58	2.6%
**AMS**	Dust	2.70	27.9%	0.59	6.8%	3.99	38.8%	6.98	34.8%	1.45	8.6%	10.38	47.3%
	SFCOA1	1.44	14.9%	3.00	34.5%	0.48	4.7%	2.47	12.3%	5.12	30.4%	0.84	3.8%
	SFCOA2	1.76	18.2%	1.52	17.5%	1.91	18.6%	2.83	14.1%	2.43	14.5%	3.07	14.0%
	NSOA	0.43	4.4%	0.36	4.2%	0.47	4.5%	0.55	2.7%	0.49	2.9%	0.59	2.7%
	SOOA	1.61	16.6%	2.54	29.2%	1.04	10.1%	2.64	13.2%	4.10	24.4%	1.75	8.0%
	WOOA	1.75	18.0%	0.69	7.9%	2.40	23.3%	2.92	14.6%	1.16	6.9%	4.00	18.2%
**Subtotal w/AMS**		9.68	100%	8.70	100%	10.29	100%	20.03	100%	16.82	100%	21.93	100%
**ACSM**	HOA							0.88	4.5%	1.05	6.5%	0.74	3.5%
	COA							0.77	3.9%	1.01	6.2%	0.58	2.7%
**EESI**	Dust-OA	1.42	14.9%	0.51	6.0%	1.97	19.4%	3.52	18.0%	1.26	7.7%	4.91	22.9%
	Carbonate	0.99	10.4%	0.29	3.4%	1.43	14.0%	1.02	5.2%	0.30	1.8%	1.47	6.9%
	Cig-OA	0.44	4.6%	0.03	0.3%	0.69	6.8%	1.43	7.3%	0.09	0.6%	2.25	10.5%
	ln-SFCOA	2.35	24.7%	4.51	53.6%	1.02	10.0%	3.66	18.7%	7.03	43.1%	1.58	7.4%
	hn-SFCOA	0.54	5.7%	0.40	4.7%	0.63	6.2%	2.17	11.1%	1.59	9.8%	2.53	11.8%
	SON	1.05	11.0%	0.57	6.7%	1.35	13.2%	1.32	6.8%	0.71	4.4%	1.69	7.9%
	SOOA	0.74	7.8%	1.48	17.6%	0.29	2.9%	1.03	5.3%	2.05	12.6%	0.40	1.9%
	WOOA	1.99	20.9%	0.65	7.7%	2.81	27.6%	3.72	19.0%	1.21	7.4%	5.26	24.5%
**Subtotal w/EESI**		9.52	100%	8.42	100%	10.19	100%	19.52	100%	16.31	100%	21.42	100%

a Here, the WSOA and OA include carbonate (10.4% and 5.2%, respectively). The water-insoluble OA sources (i.e., HOA, COA) are retrieved from ACSM source apportionment.

The major sources in Xi’an were Dust (47.3% or 29.8%, from AMS or EESI), followed by WOOA (18.2% or 24.5%), and SFC-related OAs (17.8% or 19.2%). For the first time, the contribution of carbonate and organics to dust were separately quantified with the help of EESI, revealing that a major fraction (77%) of carbonaceous dust was OA rather than carbonate. In Chongqing, SFC-related OAs including partially aged biomass burning and coal combustion emissions were the largest contributor of OA in Chongqing (44.9% or 52.9%, from AMS or EESI), followed by SOOA (24.4% or 12.6%). The water-insoluble OA sources (i.e., HOA and COA) accounted for 12.3% of OA in Chongqing, and 6.0% in Xi’an. The quantified sources with unprecedented near-molecular details not only act as a proof of the successful practice combining offline and online mass spectrometric and statistical techniques (Figure S18), but also provide valuable insights for subsequent chemical, modelling, and health studies, as well as policy making for air pollution mitigation.

## Supporting Information Available

Additional experimental details and methods; (Table S1) Overview of offline and online techniques and measurements; (Figure S1) Sulfate from EESI vs. IC; Filtering EESI ions for PMF; (Table S2, Figure S2) Quantifying carbonate carbon; (Figure S3) *Q*/*Q*_exp_; (Table S3–S4) Criteria to select bootstrapped PMF runs; (Figure S4–S5) Fitted relative standard deviation for AMS or EESI factors; (Table S5) Fitted coefficients from MLR; (Figure S6) Quantified EESI WSOA factors and carbonate; (Figure S7) Rescaling ACSM OC; (Figure S8–S11) Time series and profiles of AMS and EESI factors; (Figure S12–S13) Correlations between AMS/EESI factors, and markers; (Figure S14) Time series of AMS/EESI SOOA and temperature; (Table S6) Recoveries of AMS and EESI factors; (Table S7) Fitted fraction of SOC in each AMS factor; (Figure S15) Recovered OC and SOC; (Figure S16) Recovered OA from EESI vs. AMS; (Figure S17) Time series of recovered EESI OA sources and carbonate; (Figure S18) Schematic of data analyses of offline source apportionment in this study (PDF)
