# Supplementary material for: Composition and Sources of Organic Aerosol in Two Megacities in Western China Using Complementary Mass Spectrometric and Statistical Techniques
Source: ACS EST Air. 2024 Jul 16;1(9):1053–65. doi: 10.1021/acsestair.4c00051 (PMC11406521; doi:10.1021/acsestair.4c00051)
Supplement: Supplementary file 1 — ea4c00051_si_001.pdf [file ea4c00051_si_001.pdf]

Supporting Information to

Composition and sources of organic aerosol in two megacities in western China using complementary mass spectrometric and statistical techniques

Tianqu Cui[a*], Manousos I. Manousakas[a], Qiyuan Wang[b], Gaëlle Uzu[c], Yufang Hao[a], Peeyush Khare[a#], Lu Qi[a], Yang Chen[d], Yuemei Han[b], Jay G. Slowik[a], Jean-Luc Jaffrezo[c], Junji Cao[b], André S. H. Prévôt[a*], Kaspar R. Daellenbach[a*]

[a] PSI Center for Energy and Environmental Sciences, Paul Scherrer Institute, 5232 Villigen-PSI, Switzerland

[b] Key Laboratory of Aerosol Chemistry and Physics, Institute of Earth Environment, Chinese Academy of Sciences, Xi'an, 710061, China

[C] Institut des Géosciences de l'Environnement, CNRS, UGA, IRD, Grenoble INP, INRAE, Grenoble, 38000, France

[d] Research Center for Atmospheric Environment, Chongqing Institute of Green and Intelligent Technology, Chinese Academy of Sciences, Chongqing, 400714, China

[#] Now at: Institute for Energy and Climate Research, IEK-8: Troposphere, Forschungszentrum Jülich GmbH, Jülich, Germany

[*] Correspondence: tianqu.cui@psi.ch, andre.prevot@psi.ch, kaspar.daellenbach@psi.ch

Overview of analytical techniques

Table S1. Overview of offline and online techniques and measurements

Online or offline	Method	PM components or properties	Examples	No. of filters (or days)	Sources apportioned
Offline (filter-based)	Gravimetric	PM mass	-	316	n/a
	Elemental carbon - organic carbon (EC-OC)	EC, OC	-	316	Biomass, Coal, Dust, Fireworks, Industry, Secondary inorganic, Traffic (not reported in this study)
	Total organic carbon (TOC)	Water-soluble organic/inorganic carbon (WSOC/WSIC)	-	321	
	Ion chromatography (IC)	Water-soluble ions	K^+, Mg^{2+}, Ca^{2+}, NH_4^+, SO_4^{2-}, NO_3^-, Cl^-	316	
	Energy-dispersive x-ray fluorescence spectrometry (ED-XRF)	Elements	As, Ba, Ca, Co, Cr, Cu, Fe, K, Mn, Ni, Pb, Sc, Se, Sr, Ti, V, Zn	316	
	Inductively coupled plasma mass spectrometry (ICP-MS)	Elements	As, Ba, Ca, Co, Cr, Cu, Fe, K, Mn, Ni, Pb, Sc, Se, Sr, Ti, V, Zn	40	n/a
	High-performance liquid chromatography pulsed amperometric detection (HPLC-PAD)	Anhydro-sugars, saccharides	levoglucosan, arabitol, glucose	107	
	Liquid chromatography (electrospray ionization) mass spectrometry (LC-(ESI)-MS)	Acids	phthalic, pyruvic, vanillic acids, 3-methyl-1,2,3-butanetricarboxylic acid (MBTCA)	107	
	Gas chromatography mass spectrometry (GC-MS)	n-Alkanes, PAHs	pentadecane, acenapthene	161	
	Aerosol mass spectrometer (AMS)	Bulk OA, sulfate, nitrate, ammonium, chloride	OA fragment ions: $C_2H_4O_2^+$	310	Dust, SFCOA1, SFCOA2, NSOA, SOOA, WOOA
	Extractive electrospray ionization (EESI)	Near-molecular ions	$C_{10}H_{15}N_2^+$ (e.g., nicotine), $C_6H_{10}O_5Na^+$ (e.g., levoglucosan), $Na_2HCO_3^+$ (CO3-related)	310	Dust-OA, Cig-OA, ln-SFCOA, hn-SFCOA, SON, SOOA, WOOA
Online (real-time, in field)	-	Meteorological	temperature, RH, wind speed, wind direction	321	
	-	Gases	O_3, NO_x, SO_2	321	
	Aerosol chemical speciation monitor (ACSM)	Bluk OA, sulfate, nitrate, ammonium, chloride	OA fragment ions	169	HOA, COA, CCOA, BBOA, OOA (not all reported in this study)
	Ambient multi-metals monitor (Xact)	(trace) Elements	Fe, Zn, Pb, Cu, Ti, Ni	138	Biomass, Coal, Dust, Industry-Regional, Traffic (not reported in this study)

Correction of EESI and AMS data for sulfate and nitrate quantification

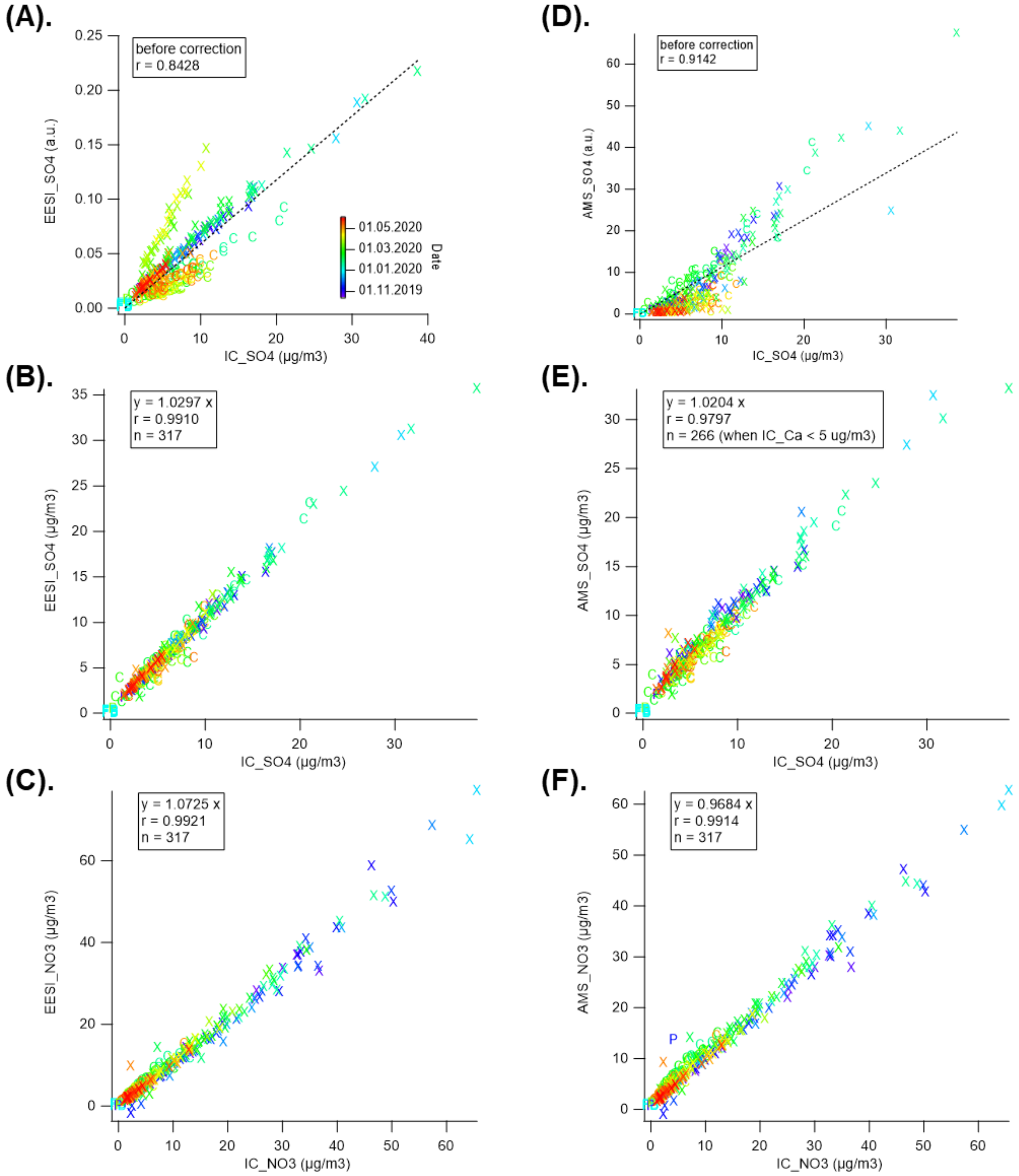

Figure S1. Sulfate and nitrate measured using EESI or AMS vs. IC. (A) Sulfate signal in EESI before the correction using the isotopically-labeled internal standard vs. IC; (B) sulfate eventually quantified using EESI vs. IC; (C) nitrate quantified using EESI vs. IC; (D) sulfate signal in AMS before the correction using the isotopically-labeled internal standard vs. IC; (E) sulfate quantified using AMS vs. IC; (F) nitrate quantified using AMS vs. IC. Marker text indicates cities (C - Chongqing, X - Xi'an, FB - field blank, P – filters excluded from PMF). Marker color represents collection date of filters.

Filtering EESI ions from PMF inputs

In order to optimize PMF inputs, a set of criteria were developed to filter out ions with relatively low signal-to-noise or sample-to-blank ratio from the 2041 ions originally fitted in Tofware.

By definition, for each ion over the 321 sample and field blank filters:

Filter_1 = (avg_sample – avg_water) / avg_water

where avg_sample is the ion intensity (normalized by intensity of Na_2I^+) averaged during the 8-min of measurement; avg_water is the ion intensity (normalized by intensity of Na_2I^+) averaged during the 12-min of water flushing. This filter aims to identify ions from contaminants present in the background of measurement, such as from the flushing water, containers, nebulizer, and instrument.

Filter_2 = (sample_filter – avg_FB) / avg_FB

where sample_filter is the semi-quantified ion concentration (µg/m3) in a sample filter; avg_FB is the semi-quantified ion concentration (µg/m3) averaged from the field blank filters (n = 3 for Chongqing and Xi'an). This filter aims to identify ions present with in the field blank filters.

Filter_3 = data_FBsubt / error_FBsubt

where data_FBsubt and error_FBsubt are the data and error matrices as the PMF inputs. This filter calculates the final signal-to-noise ratio of each ion.

The threshold was eventually determined to be the first quartile for all the filters (n = 321). In other words, the 25% worst ions according to each filter were removed from PMF inputs. By doing this, most ions with relatively high sample-to-blank and signal-to-noise ratios were kept. Actually, the resultant PMF solutions are stable with varied thresholds of these filters. The selection (or filtration) of ions only mattered whether these ions were present in the PMF factor profiles.

In addition, the primary ion related (e.g., Na_2I^+), internal standard related (e.g., $Na_2[^{15}N]O_3^+$, $Na_3[^{34}S]O_4^+$), and all other inorganic ions (e.g., $CaI(H_2O)_2^+$, $Na_2Cl(NaI)^+$) were excluded from PMF inputs for OA source apportionment. As a result, 1127 ions were retained in the PMF input that led to the final solution.

Quantifying carbonate carbon in water-soluble aerosol

Carbonate (CO3) is an important constituent in dust aerosol but not often measured along with other water-soluble ions using IC, or with WSOC using a thermal/optical carbon analyzer. For example, the widely used commercial OC-EC analyzer (e.g., the Sunset OC-EC analyzer, Sunset Labs, Tigard, OR, USA) is not equipped with an injection port for *in situ* acidification for carbonate carbon removal.

Table S2. Capability of organic carbon (OC) and carbonate carbon measured in different techniques

<table>
<tr><th></th><th>OC-EC[a]</th><th>TOC (ws)[b]</th><th>ACSM</th><th>AMS (offline, ws)</th><th>EESI (offline, ws)</th></tr>
<tr><td>Organic carbon</td><td rowspan="2">Combined[c]</td><td>Yes</td><td>Yes</td><td rowspan="2">Combined</td><td>Yes</td></tr>
<tr><td>Carbonate carbon</td><td>Yes</td><td>No</td><td>Yes</td></tr>
</table>

[a] The OC-EC measurement was conducted without acidification.

[b] ws – water-soluble

[c] Both organic carbon (OC) and carbonate carbon were measured, but reported as one quantity. The OC-EC measurement was conducted without acidification.

In this study, carbonate carbon from all filters (n = 321) was measured using EESI-LTOF. Filter extracts from a smaller number of selected filters (n = 18) were used to measure carbonate carbon (or water-soluble inorganic carbon, WSIC) using the Shimadzu TOC analyzer, to determine the response factor from the EESI-measured CO3-related ion (e.g., $Na_2HCO_3^+$, $Na_3CO_3^+$, $Na_2HCO_3(NaI)^+$, $Na_3CO_3(NaI)^+$) intensity to the concentration of carbonate carbon (μg mL^{-1}). $Na_2HCO_3^+$ correlates best with WSIC (Figure S2) and was thus used to quantify carbonate carbon via EESI measurements.

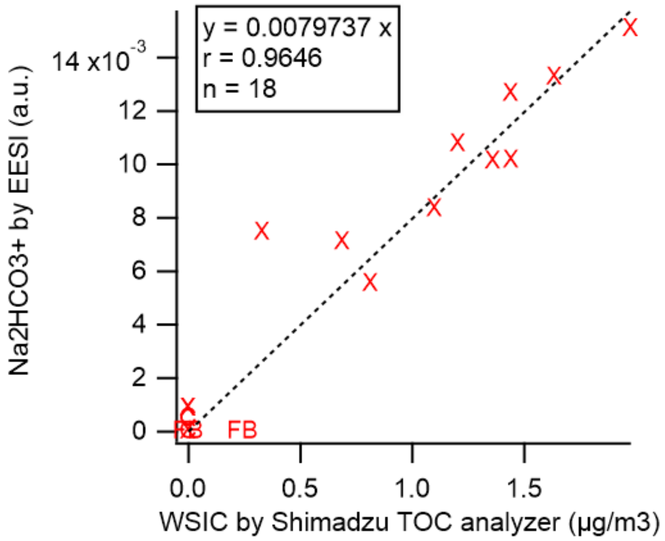

Figure S2. An EESI CO3-related ion ($Na_2HCO_3^+$) semi-quantified using $Na_3[^{34}S]O_4^+$ as internal standard vs. WSIC measured by Shimadzu TOC analyzer for selected filter samples (C-Chongqing, X-Xi'an; FB-field blank).

PMF for offline AMS and EESI data: constraints, bootstrap, criterion-based selection, and uncertainties

1. Application of constraints

For AMS:

Initially, six-factor solutions were resolved with the best level of interpretation without any constraints. However, some previously-reported factors in Xi'an, such as hydrocarbon-like OA (HOA), cooking OA (COA), and coal combustion OA (CCOA), were not resolved. Attempts to resolve these less-water-soluble sources were made by using published reference profiles separately as constraints in PMF. These reference profiles include:

(i) A high-resolution (HR) HOA profile (Elser et al., 2016), in combination with a unit-mass-resolution (UMR) HOA profile (Crippa et al., 2013).

(ii) Eight online and offline (water-soluble) HR AMS CCOA profiles (Xu et al., 2020).

However, the resulted HOA or CCOA factors were not satisfying, because (1) their time series was largely discrete, with most days being zero; (2) their time series was not strongly correlated with the same factor from ACSM. Therefore, the solutions with constrained HOA or CCOA were not accepted, likely because of the low water-solubility of the OA constituents.

For these water-insoluble factors, we relied instead on HOA and COA from ACSM, and identified CCOA to be one of the emission sources affecting the solid fuel combustion (SFC)-related OA factors (SFCOAs).

For EESI:

To keep good resolved factors and to guide PMF to explore solution space, a profile identified as summer oxygenated OA (SOOA) from an 18-factor solution was retrieved and used as a constraint (a-value = 0.05) to resolve this SOOA factor from solutions with fewer factors. A profile (later named SON) strongly correlating with an AMS nitrogen- and sulfur-containing OA (NSOA) from an 11-factor solution was obtained as a constraint (a-value = 0.05) for such purposes. These two constrained profiles, SOOA and SON, can be found in the final solution.

2. Q/Q_{exp}

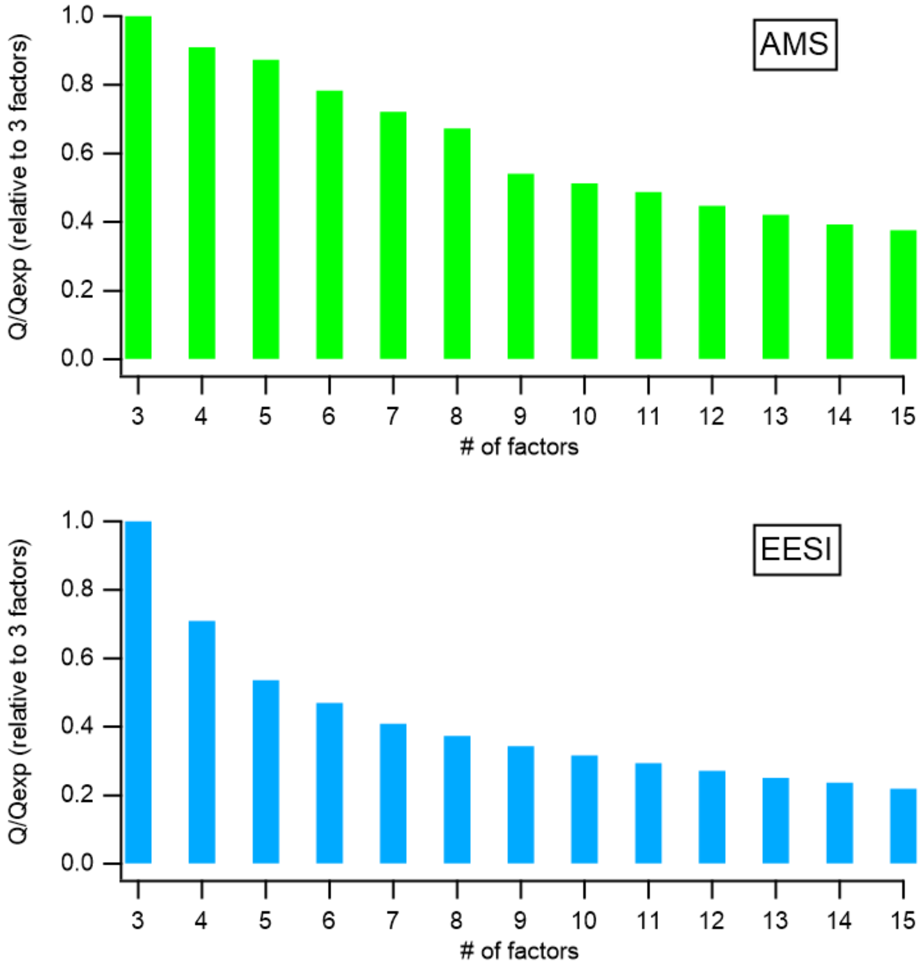

Figure S3. Q/Q_{exp} as a function of number of AMS (top) or EESI (bottom) factors.

3. Bootstrap, criterion-based selection, and uncertainties

After 1000 bootstrapped runs, a criterion-based selection was performed to select a number of solutions to estimate uncertainties. The criteria are set up based on the sorted correlation of time series with the base-case solution. The threshold was determined at an inflection point on the descending curve of Pearson correlation coefficient (r).

For AMS:

Table S3. Criteria of selected solutions from bootstrapped AMS PMF runs

#	Factors	Criteria	Pearson r
1	Dust	Same factor in base case	0.969
2	SFCOA1	Same factor in base case	0.986
3	SFCOA2	Same factor in base case	0.980
4	NSOA	Same factor in base case	0.963
5	SOOA	Same factor in base case	0.956
6	WOOA	Same factor in base case	0.977

As a result, 698 out of the 1000 bootstrapped runs were selected according to the criteria above. The uncertainties over these selected runs are reported as relative standard deviation (RSD), and an absolute uncertainty. Both were fitted using the following equation:

$$\sigma_{PMF} = \sqrt{(RSD \times m_{PMF})^2 + {\sigma_{abs}}^2}$$

where σ_{PMF} = standard deviation of a factor at a time point in bootstrapped solutions, RSD = relative standard deviation to be fitted, m_{PMF} = mean of a factor at a time point over bootstrapped solutions, σ_{abs} = an absolute uncertainty to be fitted, accounting for non-PMF-related uncertainties, such as detection limit.

The fitted RSD is from 6.6%-13.4%, and the fitted absolute uncertainty is below 1.0e-3 (Figure below), indicating quite small relative uncertainties over the bootstrapped runs especially at larger concentrations (> 1 µg m^{-3}).

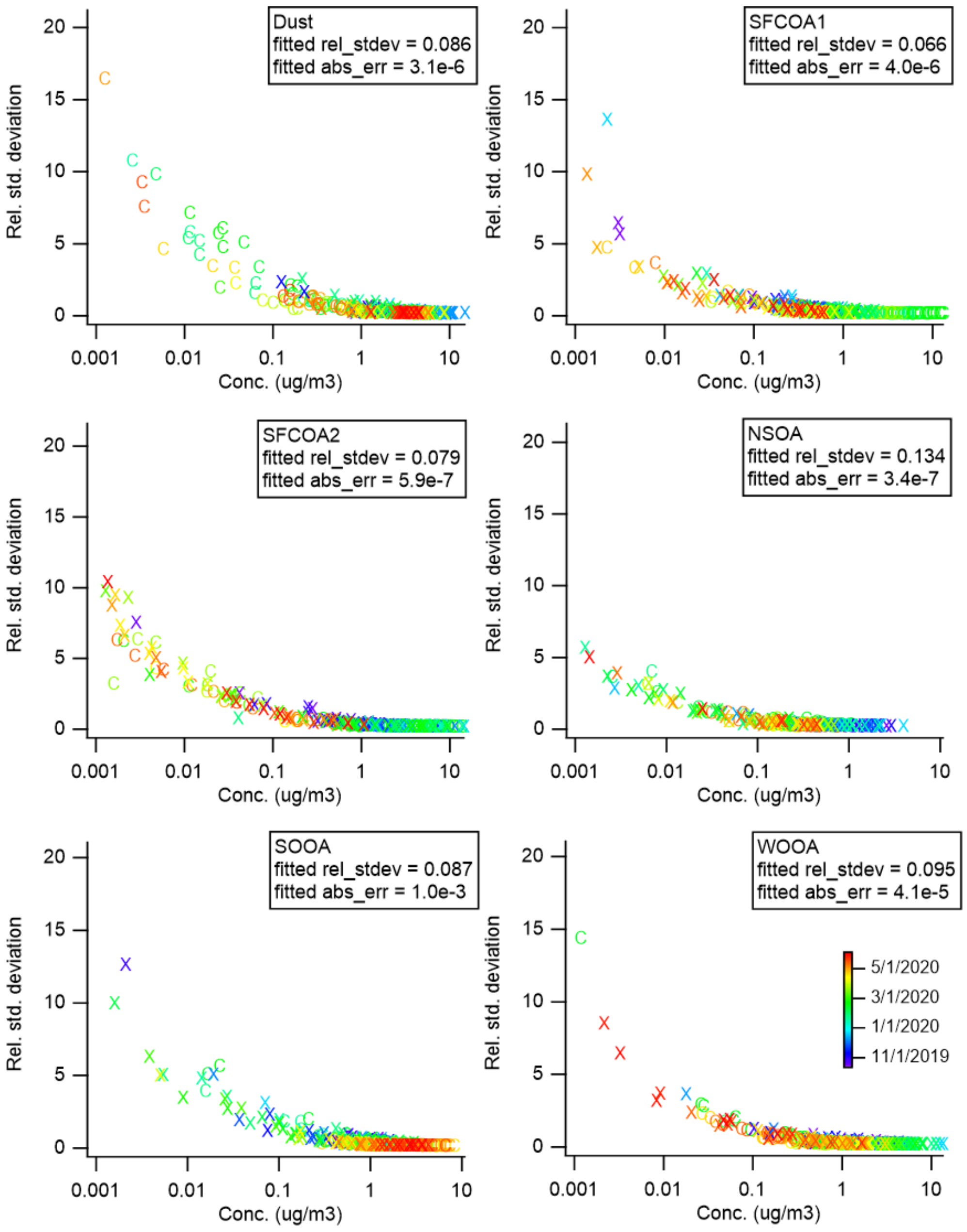

Figure S4. Fitted relative standard deviation (as well as absolute uncertainty) as a function of AMS WSOA factor concentration (μg m^{-3}). Marker (C-Chongqing, X-Xi'an) colors represent collection date of filters.

For EESI:

Table S4. Criteria of selected solutions from bootstrapped EESI PMF runs

#	Factors	Criteria	Pearson r	Uncentered r
1	Dust	Same factor in base case	0.9161	
2	Cig-OA	Same factor in base case	0.9478	0.9743
3	ln-SFCOA	Same factor in base case	0.92	
4	hn-SFCOA	Same factor in base case	0.9861	
5	SON	n/a (profile constrained)		
6	SOOA	n/a (profile constrained)		
7	WOOA	Same factor in base case	0.9775	

As a result, 259 out of the 1000 bootstrapped runs were selected according to the criteria above. The unselected factor typically has a Cig-OA factor different from those selected. The uncertainties over these selected runs are also reported as relative standard deviation (RSD) and an absolute uncertainty. Both were fitted using the same equation as for AMS.

The fitted RSD is between 4.0%-10.9%, and the fitted absolute uncertainty is 0.0145 or below (Figure below), indicating quite small relative uncertainties over the bootstrapped runs especially at larger concentrations (> 1 μg m^{-3}).

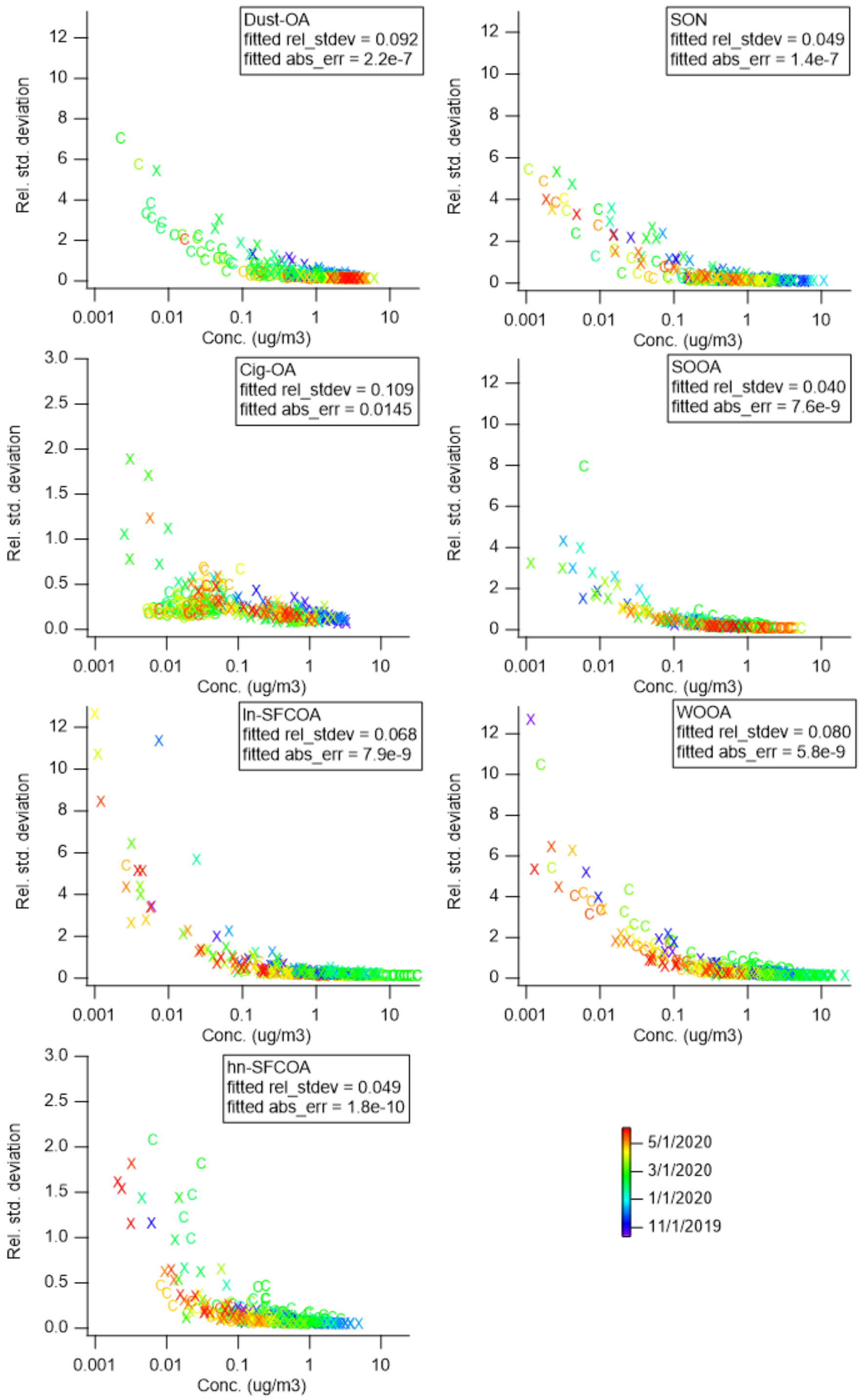

Figure S5. Fitted relative standard deviation (as well as absolute uncertainty) as a function of EESI WSOA factor concentration (μg m^{-3}). The quantification of EESI WSOA is described in later sections. Marker (C-Chongqing, X-Xi’an) colors represent collection date of filters.

Quantifying EESI WSOA factor and carbonate

To convert the EESI PMF factors to water-soluble concentrations in µg m^{-3}, time series of the PMF-resolved EESI factors, along with carbonate carbon, were regressed to the time series of [WSOA + carbonate] = (WSOC + WSIC) × (OA:OC) in each sample, using a multiple linear regression (MLR) model on a Bayesian statistical platform implemented in Python (PyStan).

The fitted coefficients are listed in the Table below. Here, the fitted coefficients reflect relative sensitivities of the semi-quantified WSOA factors: the smaller the coefficient, the more sensitive is the EESI to the factor. The EESI is most sensitive to hn-SFCOA and Cig-OA, probably due to the $C_xH_yN_2$ compounds (e.g., nicotine) of relatively high sensitivity in EESI. The coefficient of carbonate = 4.63, is fairly close to the OA: OC ratio of carbonate (~5).

The quantified WSOA and carbonate concentrations after applying these fitted coefficients agrees well (slope = 0.97, r = 0.98) with the quantity of measured [WSOA + carbonate], as shown in the Figure below.

Table S5. Fitted coefficients from MLR, when fitting EESI factor from signal to ambient concentration of [WSOA + carbonate] (µg m^{-3}).

**Factor**	**Fitted coef.**
Dust	0.0253
Cig-OA	0.00989
ln-SFCOA	0.0214
hn-SFCOA	0.00369
SON	0.0323
SOOA	0.0129
WOOA	0.0223
Carbonate	4.63

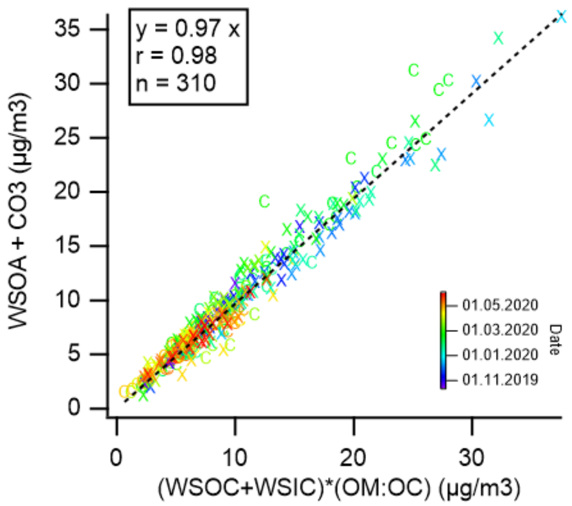

Figure S6. Sum of quantified EESI WSOA factors and carbonate concentrations as a function of [WSOA + carbonate] = (WSOC + WSIC) × (OA:OC). Marker (C-Chongqing, X-Xi'an) colors represent collection date of filters.

Parameterization of ACSM factors:

The OA factors from the ACSM are: HOA, COA, BBOA, CCOA, and oxygenated OA (OOA). In Xi'an, OOA = less-oxidized OA (LO-OOA) + more-oxidized (MO-OOA). In order to estimate OC from water-insoluble sources (WIOC), two ACSM factors are considered as water-insoluble sources: HOA, COA. A series of parameterizations are performed to make WIOC comparable with filter-based OC and WSOC from offline measurements.

(1) OC estimation

In order to derived OC of the ACSM factors, O:C and OA:OC ratios were estimated using the parametrizations in (Canagaratna et al., 2015), where:

O:C = 0.079 + 4.31*f44

OA:OC = 1.17 + 1.29*O:C

Note that the f44 (fraction of m/z 44) is not constant, since the ACSM solution was obtained from rolling PMF (i.e., dynamic profiles) with a 14-day window.

Then OC = OA / (OA:OC) was calculated for each factor at each time point.

(2) Averaging to 24-h

The high-resolution (30-60 min) ACSM OC data were averaged to a nearly 24-h time resolution, corresponding to the filter collection start and end time.

(3) Correction of RIE*CE for HOA and COA

As pointed out and suggested by (Katz et al., 2021; Xu et al., 2018), relative ionization efficiency (RIE) can be largely underestimated for factors with low carbon oxidation state, such as HOA and COA. Therefore, an increased response factor, in which relative ionization efficiency × collection efficiency or RIE × CE=2 as suggested for sites impacted by fresh sources, instead of 1.4×0.5=0.7 as default, was applied to the HOA and COA in this study. As a result, HOA and COA (as well as HOC and COC) were reduced by a factor of 2/0.7=2.86 after correction.

(4) Rescaling to $OC_{filter,\ PM2.5}$

In order to compare ACSM OC with those measured by offline techniques, the OC corrected for RIE × CE was then rescaled to OC measured by OC-EC for the $PM_{2.5}$ filters. In addition, since the ACSM in Xi'an used a PM_1 lens, it also converted OC from PM_1 factors to $OC_{filter,\ PM2.5}$. The rescaling factors were retrieved from the scatter plots of summed corrected ACSM OC as a function of $OC_{filter,\ PM2.5}$ (see the

figure below). For Chongqing (CHQ), OC_ACSM = 1.4233 * $OC_{filter,\ PM2.5}$ when both measurements are available (n = 73), while for Xi'an, OC_ACSM = 1.1422 * $OC_{filter,\ PM2.5}$ obtained only on low-dust days (AMS-Dust < 2 µg m^{-3}, n = 36), since OC measured by OC-EC include inorganic carbon from carbonates while ACSM does not.

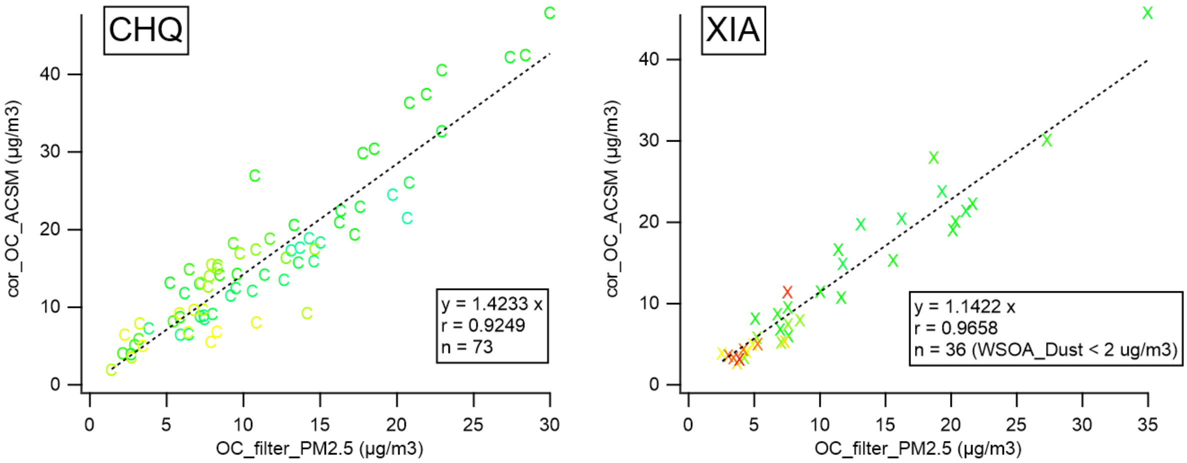

Figure S7. rescaling corrected ACSM OC to OC measured by OC-EC for $PM_{2.5}$ filters ($OC_{filter,\ PM2.5}$) in Chongqing (CHQ, left) and Xi'an (XIA, right).

AMS 6-factor solution of [WSOA+carbonate]

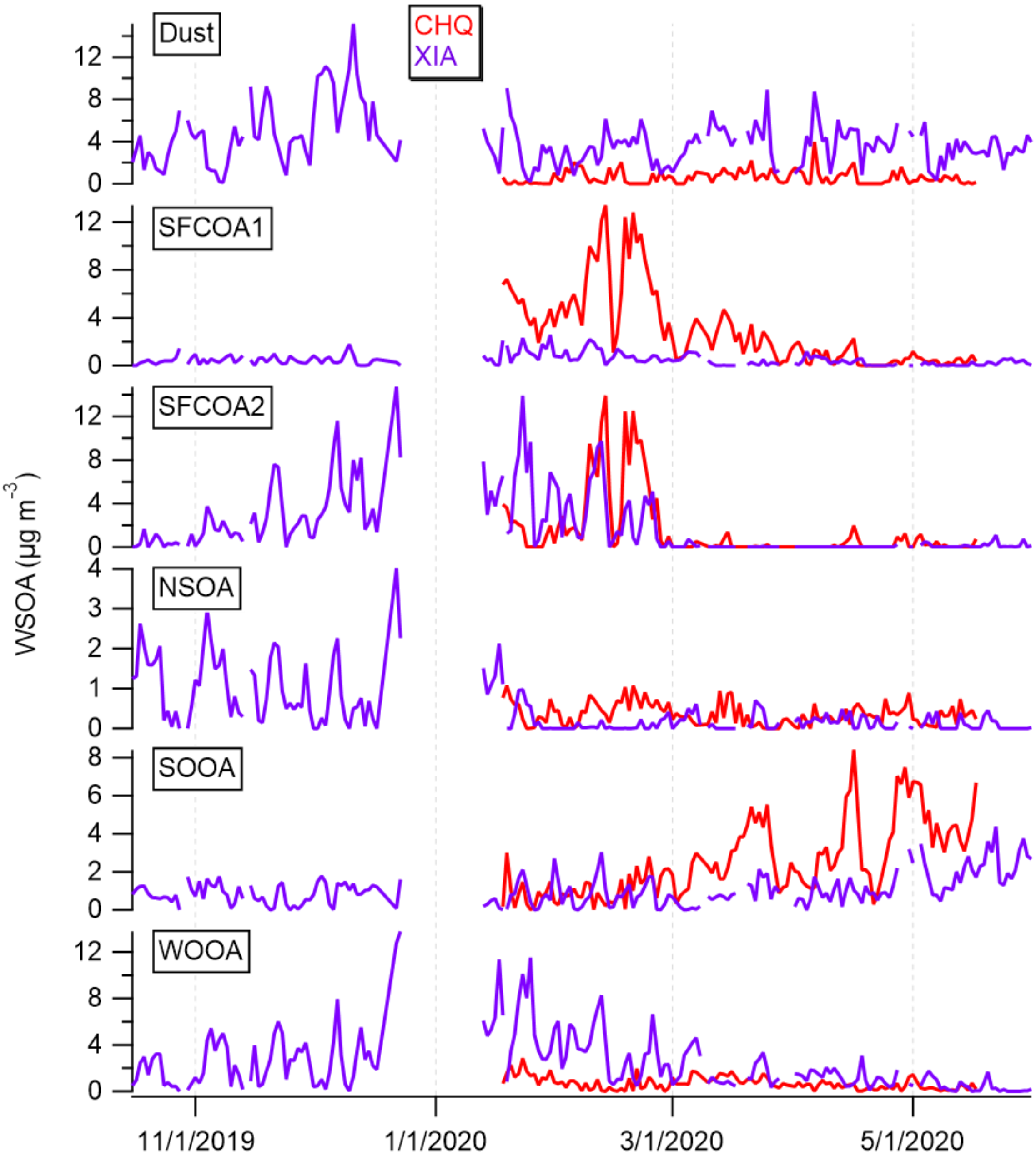

Figure S8. Time series of the six offline AMS WSOA factors in Chongqing (CHQ, red) and Xi'an (XIA, purple). SFCOA1 and SFCOA2 are two solid fuel combustion-related OAs; NSOA is nitrogen- and sulfur-containing OA; SOOA is summer oxygenated OA; WOOA is winter oxygenated OA. The Dust factor contains carbonates in addition to organics.

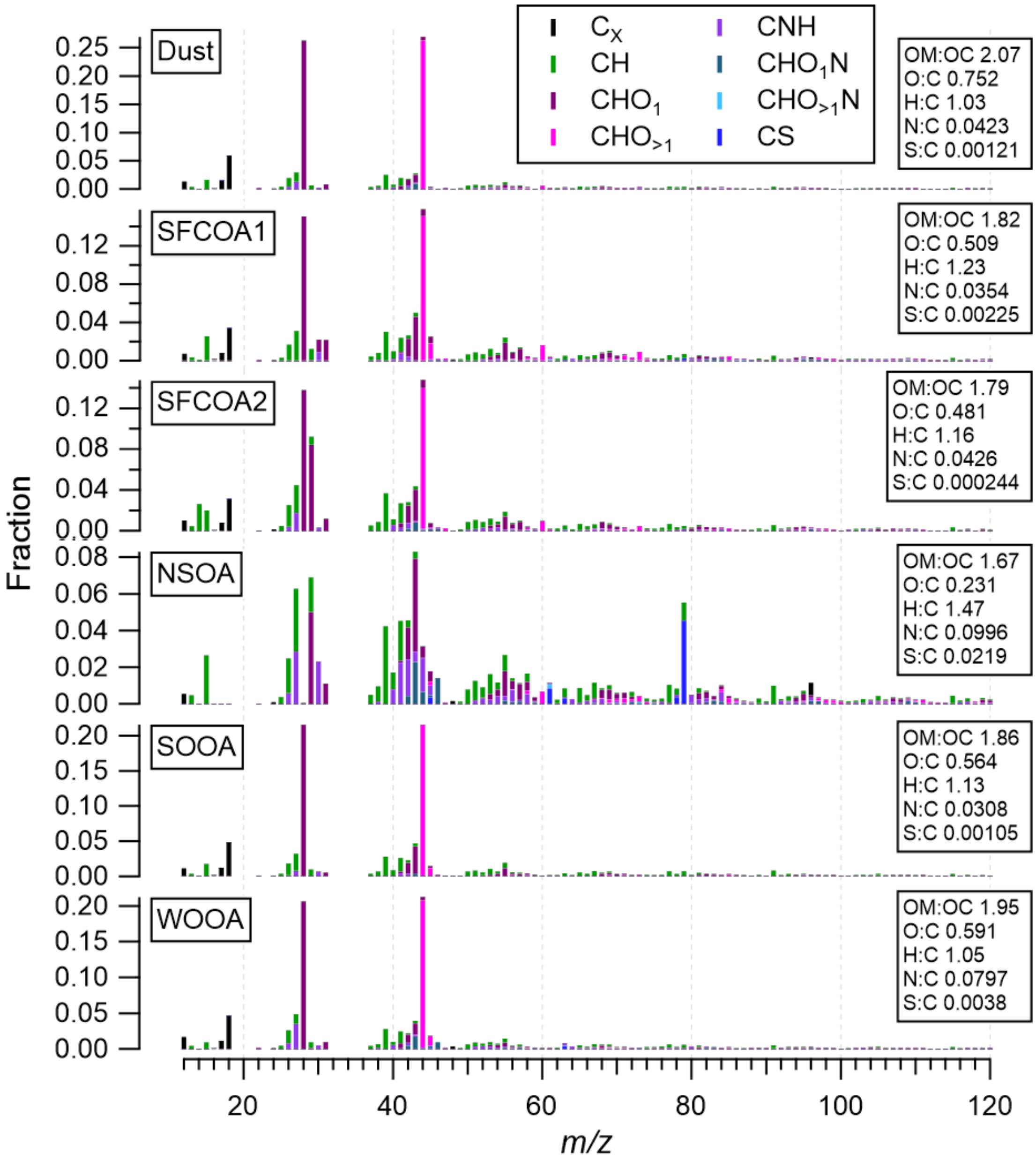

Figure S9. Profiles of the six offline AMS WSOA factors. The colors indicate ion families shown in legend. Elemental ratios of each factor are listed in respective labels. The Dust factor includes carbonate in addition to organics.

EESI 7-factor solution of WSOA

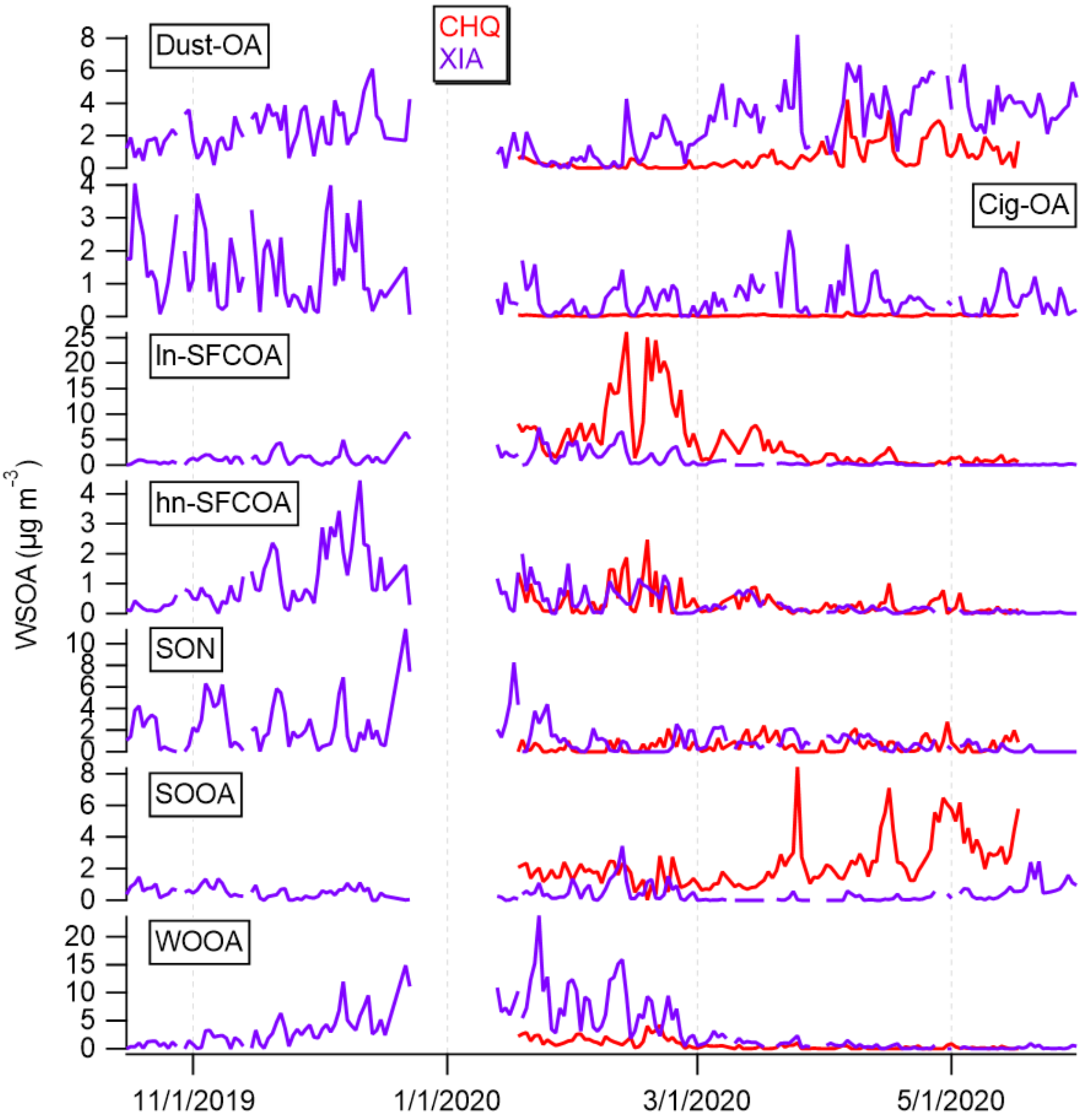

Figure S10. Time series of the seven offline EESI WSOA factors in Chongqing (CHQ, red) and Xi'an (XIA, purple). Cig-OA is cigarette-related OA; ln-SFCOA is low-nitrogen solid fuel combustion-related OA; hn-SFCOA is high-nitrogen solid fuel combustion-related OA; SON is secondary organic nitrogen; SOOA is summer oxygenated OA; WOOA is winter oxygenated OA. The EESI Dust-OA factor does not contain carbonates.

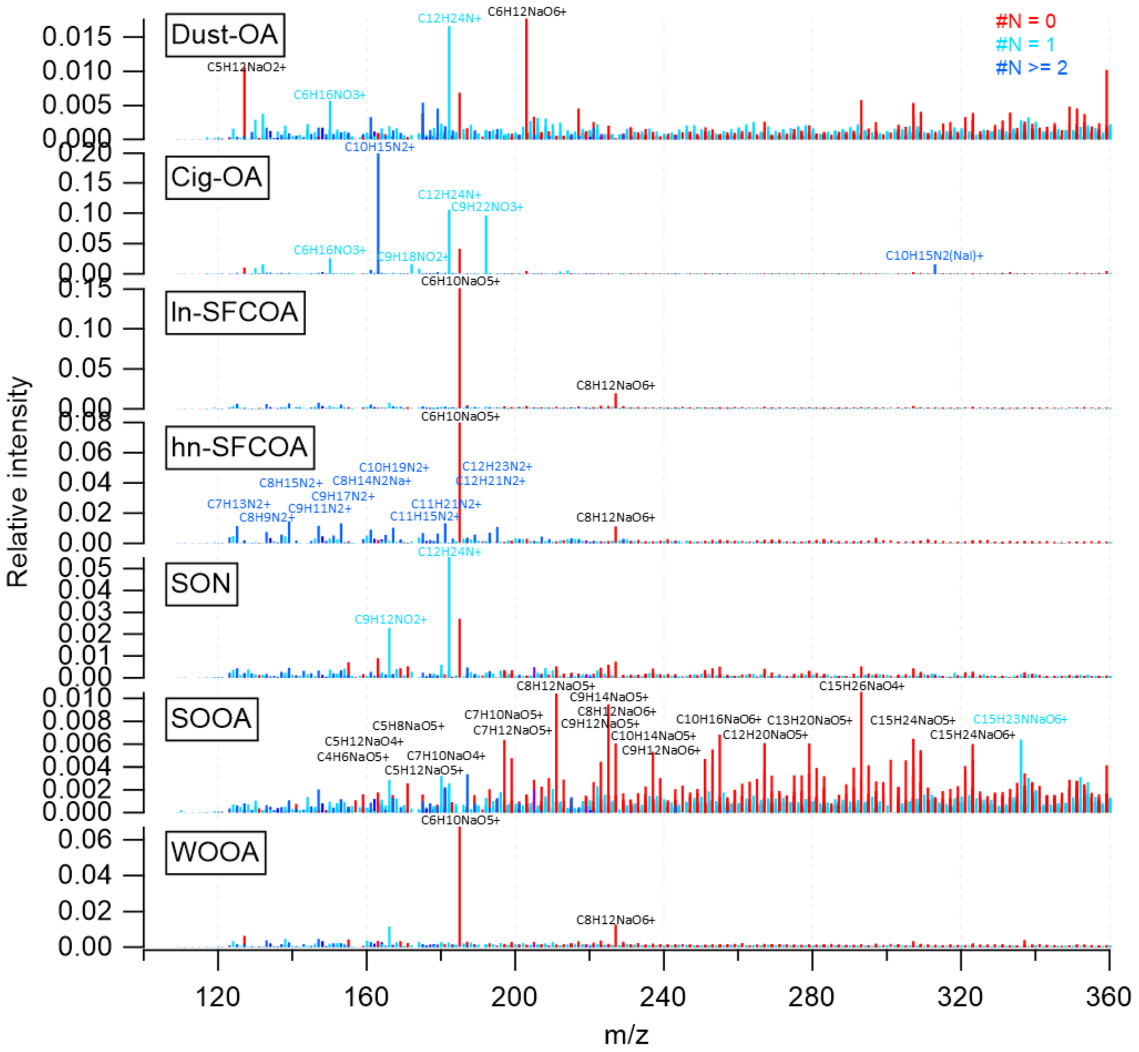

Figure S11. Profiles of the seven offline EESI WSOA factors. Molecular ions are Na^+ or H^+ adducts, colored by number of nitrogen atoms. Cig-OA is cigarette-smoking OA; ln-SFCOA is low-nitrogen solid fuel combustion-related OA; hn-SFCOA is high-nitrogen solid fuel combustion-related OA; SON is secondary organic nitrogen; SOOA is summer oxygenated OA; WOOA is winter oxygenated OA. The EESI Dust-OA factor does not contain carbonates.

Correlations between water-soluble sources from AMS, EESI, and corresponding marker

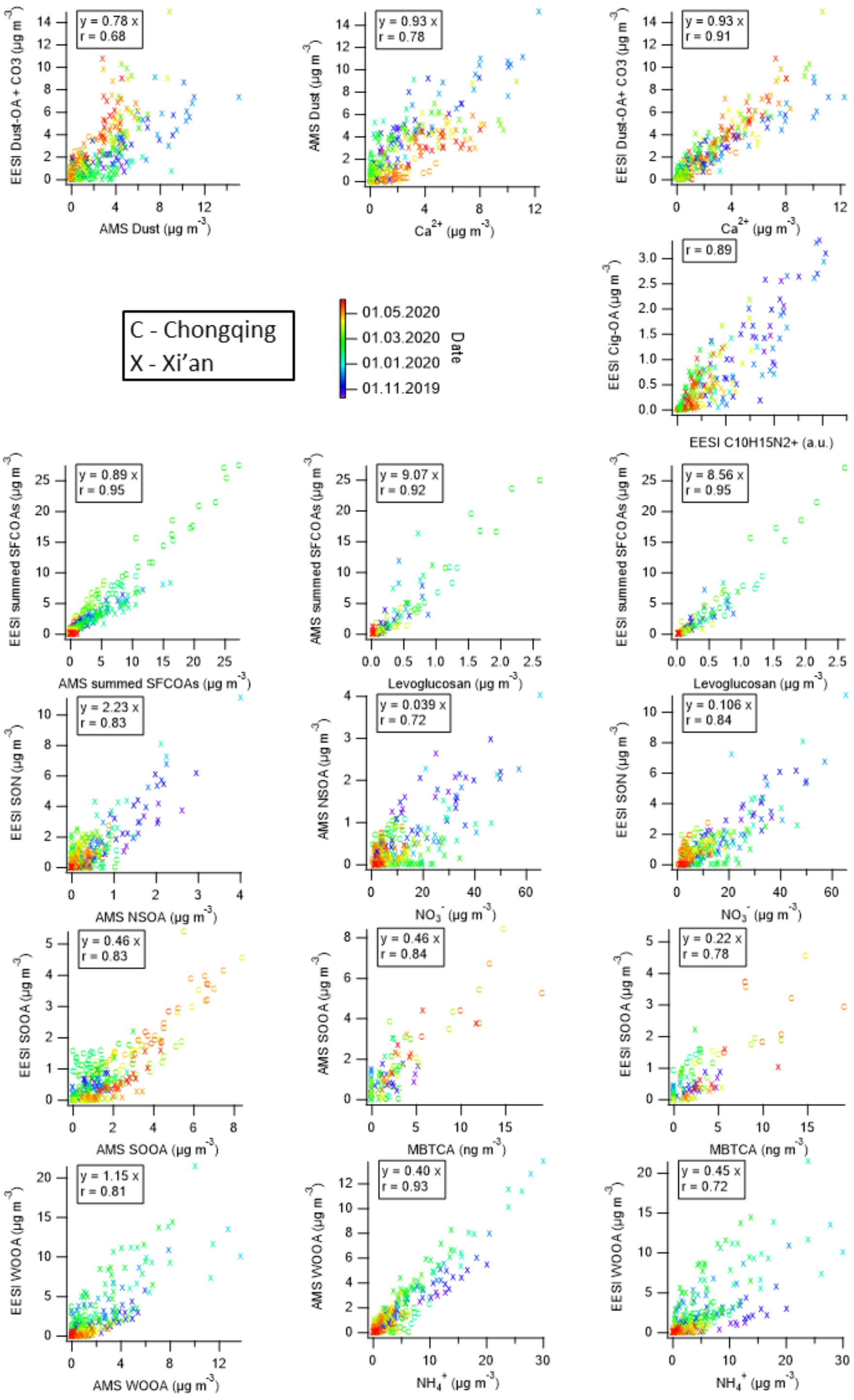

Figure S12. Scatter plots and Pearson correlations between water-soluble sources from AMS, EESI, and respective markers. From left to right: EESI vs. AMS, AMS vs. marker, and EESI vs. marker. EESI summed SFCOAs = ln-SFCOA + hn-SFCOA. AMS summed SFCOAs = SFCOA1 + SFCOA2. Ca^{2+}, NO_3^-, and NH_4^+ ware measured using IC. Levoglucosan and MBTCA were using HPLC-PAD and LC-ESI-MS, respectively (Table S1).

Correlations between all factors and species from AMS, EESI, and collocated measurements

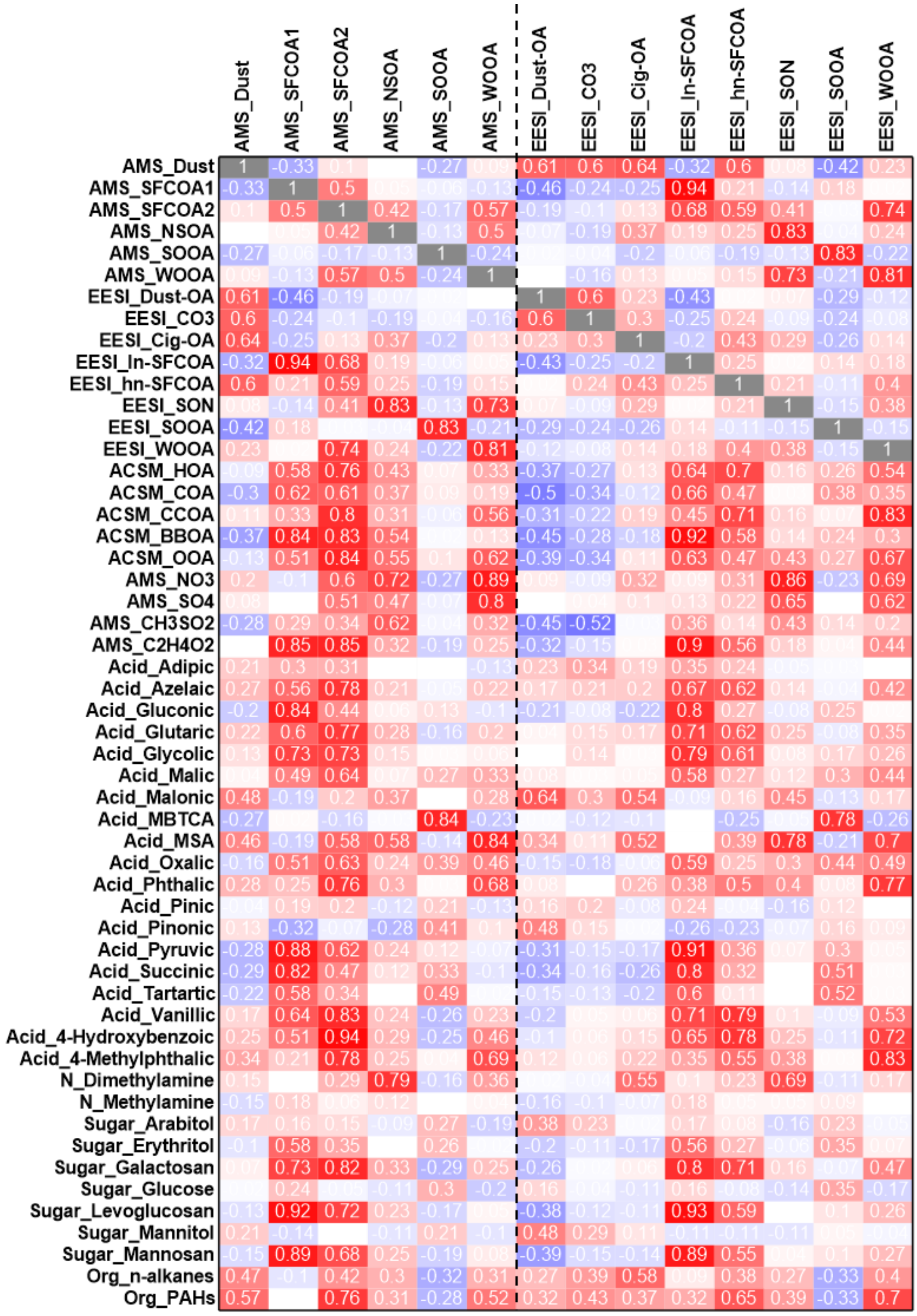

Figure S13 (a). Pearson correlation coefficients (r) among factors and species from AMS, EESI, and collocated measurements. These collocated techniques are summarized in Table S1. EESI_CO3 is quantified carbonate using carbonate-related ion(s) measured by EESI.

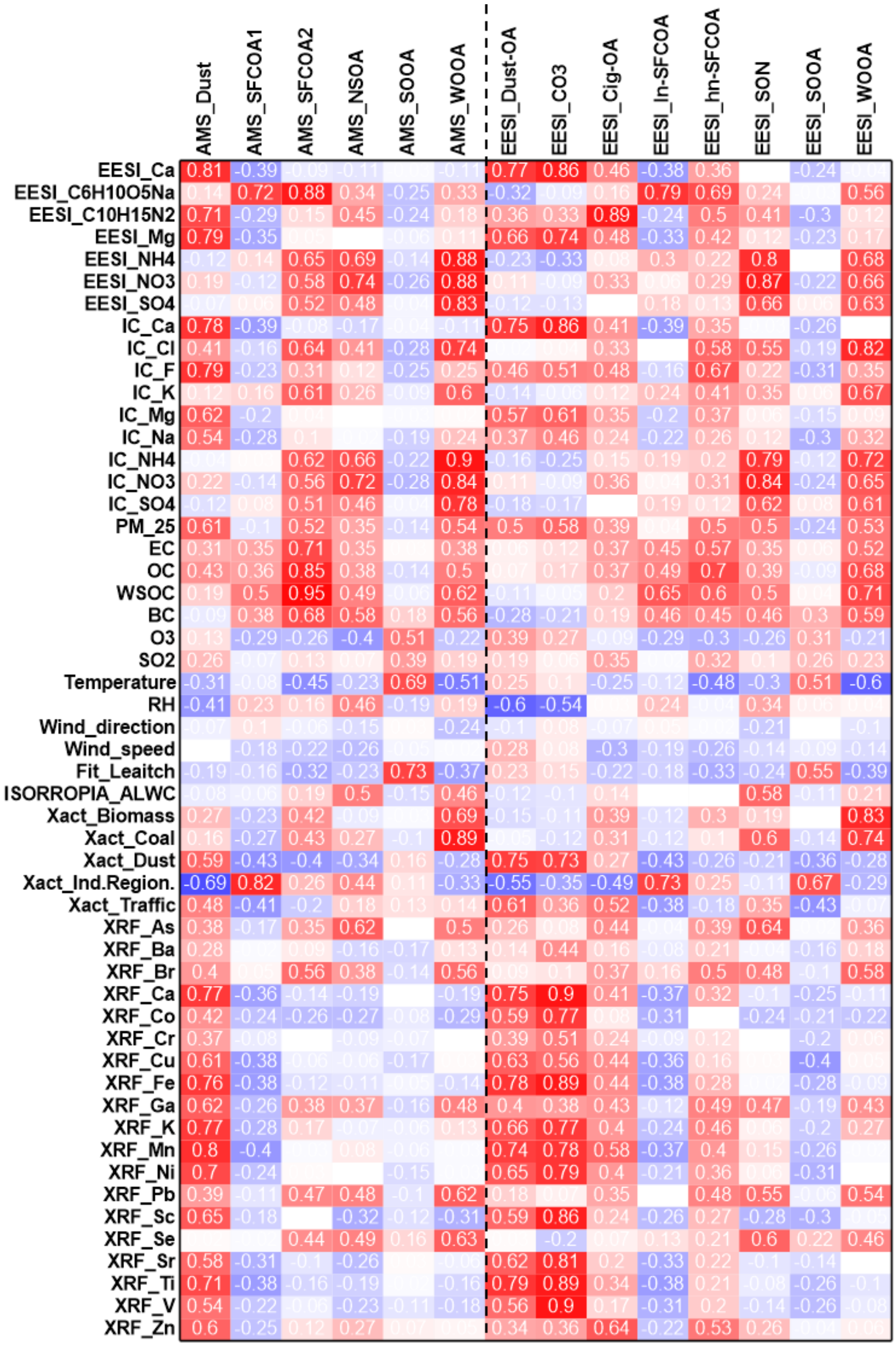

Figure S13 (b). Pearson correlation coefficients (r) among factors and species from AMS, EESI, and collocated measurements. These collocated techniques are summarized in Table S1. Fit_Leaitch is fitted SOOA using temperature data (Leaitch et al., 2011). ISORROPIA_ALWC is aerosol liquid water content estimated using ISORROPIA II (Fountoukis and Nenes, 2007).

BBOA tracers, temperature, and SOOA

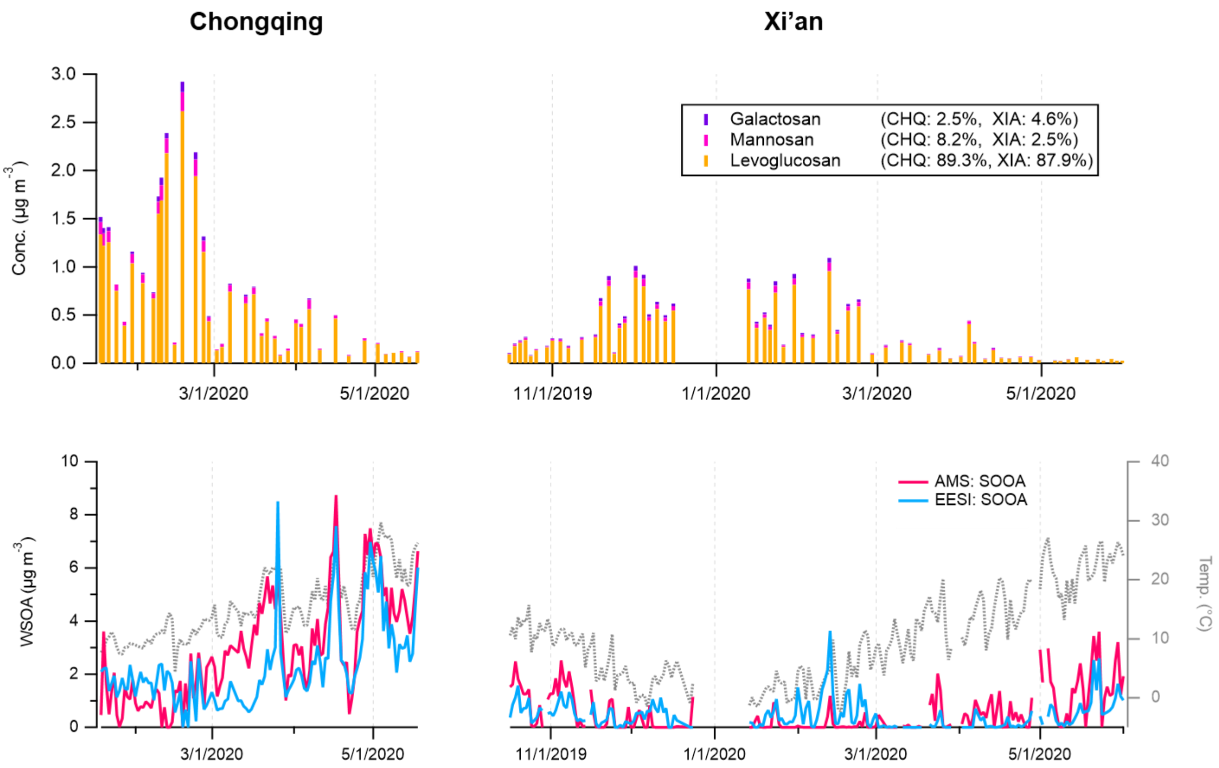

Figure S14. Time series of levoglucosan, mannosan, and galactosan (top), and AMS- & EESI-SOOAs, and temperature (bottom) in Chongqing (left) and Xi'an (right).

Recoveries from WSOA to OA

Recoveries (i.e., water-solubility) of the offline AMS WSOA factors were assessed using a multiple linear regression (MLR) model to fit their contributions to OA mass concentrations, using the following equation:

$$\mathrm{OC_{filter}} = \sum coef_k \times \frac{WSOA_k}{(\frac{OA}{OC})_k} + WSIC$$

where OC_{filter} is the OC (µg m^{-3}) concentration of the $PM_{2.5}$ filter from OC-EC measurement; k is the factor index (from 1-6 for AMS, 1-7 for EESI); $coef_k$ is the recovery coefficient (constrained from 1-10, or 10-100% water solubility) to be fitted for Factor k; $WSOA_k$ is the quantified WSOA concentration (µg m^{-3}) of Factor k; OA/OC is the OA-to-OC ratio of Factor k calculated by SoFi, WIOC = HOC + COC, is the WIOC obtained from ACSM factors (n = 168). The OC from all ACSM factors have been rescaled to OC_{filter}, and the HOC and COC (OC in HOA and COA factors) have been corrected for RIE and CE, as described in the previous section.

The $coef_k$ were determined by taking the median value of the resulting distribution over all valid MLR runs. Solubility of each factor = 1 / $coef_k$, and is shown in the table below:

Table S6. Recoveries of offline AMS and EESI factors determined using MLR.

**AMS**		**EESI**	
**Factor**	**Fitted solubility**	**Factor and CO3**	**Fitted solubility**
Dust	37.8%	Dust-OA	40.2%
SFCOA1	59.0%	ln-SFCOA	64.2%
SFCOA2	62.6%	hn-SFCOA	25.0%
NSOA	79.4%	SON	79.5%
SOOA	61.0%	SOOA	71.9%
WOOA	60.0%	WOOA	53.5%
		Cig-OA	30.7%
		carbonate (CO3)	97.2%

Once recovery rates were obtained, the WSOC in AMS factor concentrations can be recovered to OC by dividing their own solubility. The recoveries from WSOC to OC achieved good OC mass closure, as the recovered OC agreed well (slope = 0.94, r = 0.95) with the measured OC, shown in the figure below.

In addition, the secondary OC (SOC) in the AMS factors can be fitted to the ACSM OOC using a simple MLR model:

$$\text{ACSM OOC} = a*\text{SFCOC1} + b*\text{SFCOC2} + c*\text{NSOC} + d*\text{SOOC} + e*\text{WOOC}$$

where a, b, c, d, and e are coefficients to be fitted with an upper limit = 1, reflecting fraction of SOC in each factor. SFCOC1 is OC in SFCOA1 factor, similar for the other factors. Dust is assumed to be 100% primary and thus excluded in this fitting. The fitted coefficients are listed in the Table below.

Table S7. Fitted fraction of Secondary OC (SOC) in each AMS factor

AMS Factor	Fitted fraction of SOC	Comments
Dust	n/a	Assumed to be non-secondary
SFCOA1	37.0%	Upper limit = 1
SFCOA2	42.4%	Upper limit = 1
NSOA	100.0%	Upper limit = 1
SOOA	97.7%	Upper limit = 1
WOOA	100.0%	Upper limit = 1

As a result, the fitted SOC also agreed well with the corrected OOC from ACSM (slope = 0.96, r = 0.94), as shown below.

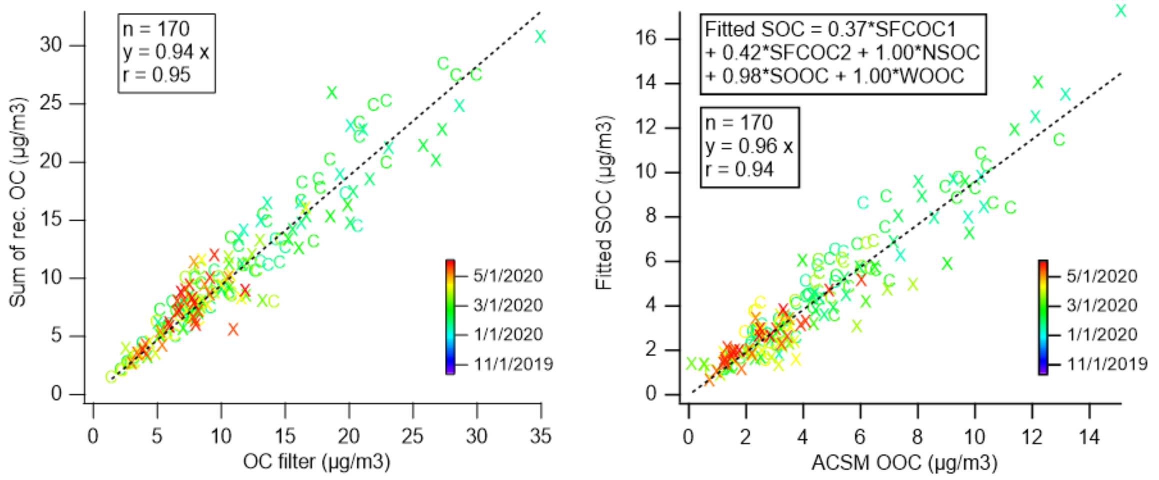

Figure S15. Recovered OC as a function of measured OC (left). Fitted secondary OC (SOC) as a function of corrected OC from ACSM OOA (right). Colors of markers (C-Chongqing, X-Xi'an) show collection date of filters. Note that the ACSM data is available only in ~55% of the dates (170 days).

The EESI water-soluble factors and carbonate were regressed to sum of the recovered AMS OA = ∑recovered OC * (OA:OC). The fitted recoveries of EESI after converted to solubility are also listed in the Table above. The same (or similar) factors between AMS and EESI have very similar solubility. For example, Dust = 37.8% in AMS and 40.2% in EESI. AMS SFCOA1 = 59.0%, while EESI ln-SFCOA = 64.2%. AMS NSOA = 79.4%, while EESI SON = 79.5%, as well as for SOOA (61.0% vs. 71.9%) and WOOA (60.0% vs. 53.5%). Carbonate is fitted to be almost totally water-soluble (97.2%). The summed recovered EESI OA and carbonate from water-soluble sources agree nicely with those from AMS, as shown in the Figure below.

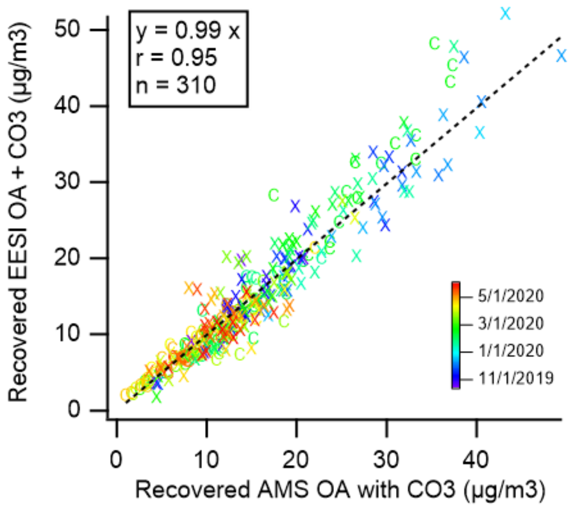

Figure S16. sum of recovered EESI OA factors and carbonate as a function of summed recovered AMS factors (including carbonate). Marker (C-Chongqing, X-Xi'an) colors represent collection date of filters.

Time series of recovered EESI OA sources and carbonate

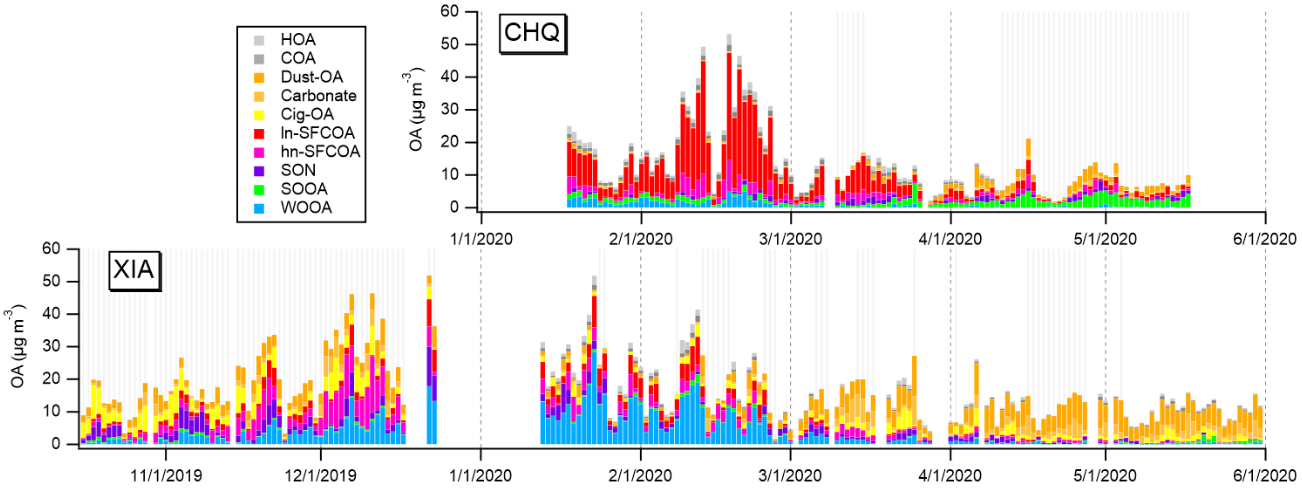

Figure S17. Recovered OA sources and carbonate in Chongqing (CHQ) and Xi'an (XIA) using EESI and ACSM. HOA and COA from ACSM data are not available in ~45% of the days marked by the vertical light grey lines.

Schematic of data analyses

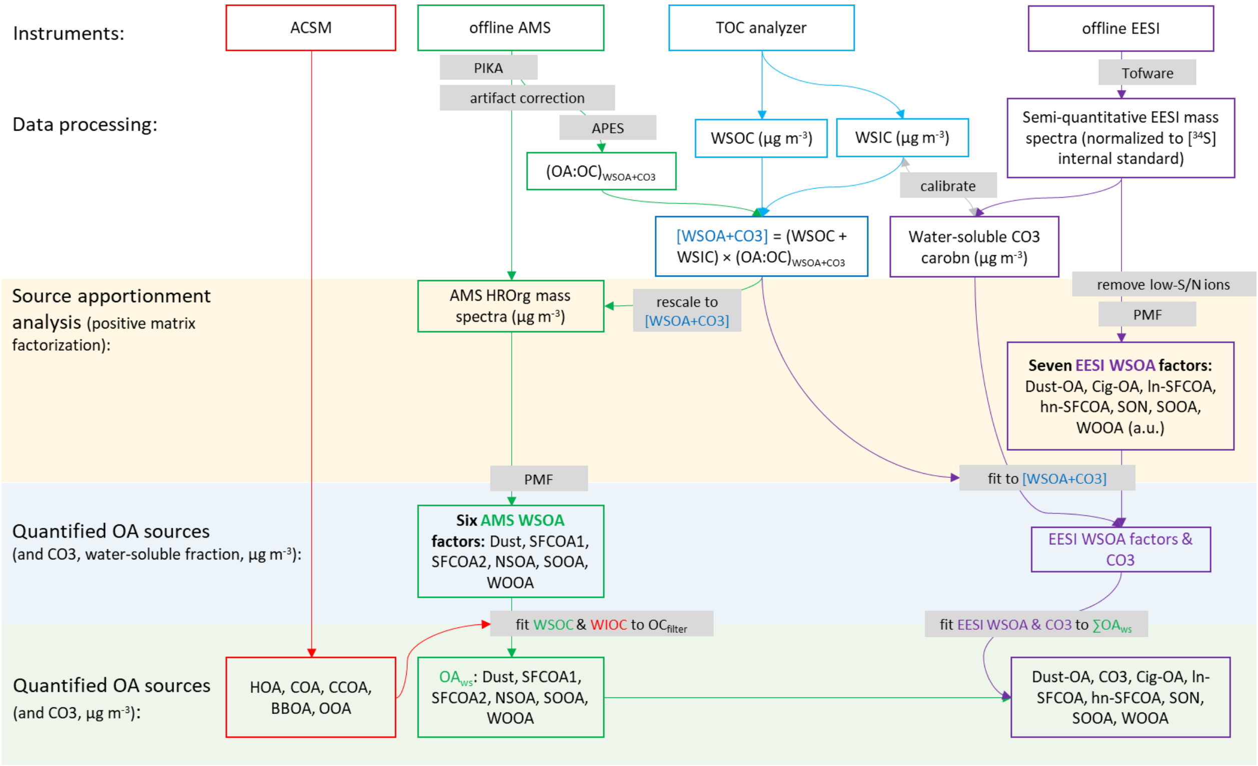

Figure S18. Schematic of data analyses of offline source apportionment in this study.

Reference

Canagaratna, M.R., Jimenez, J.L., Kroll, J.H., Chen, Q., Kessler, S.H., Massoli, P., Hildebrandt Ruiz, L., Fortner, E., Williams, L.R., Wilson, K.R., Surratt, J.D., Donahue, N.M., Jayne, J.T., Worsnop, D.R., 2015. Elemental ratio measurements of organic compounds using aerosol mass spectrometry: characterization, improved calibration, and implications. Atmos. Chem. Phys. 15, 253–272. https://doi.org/10.5194/acp-15-253-2015

Crippa, M., DeCarlo, P.F., Slowik, J.G., Mohr, C., Heringa, M.F., Chirico, R., Poulain, L., Freutel, F., Sciare, J., Cozic, J., Di Marco, C.F., Elsasser, M., Nicolas, J.B., Marchand, N., Abidi, E., Wiedensohler, A., Drewnick, F., Schneider, J., Borrmann, S., Nemitz, E., Zimmermann, R., Jaffrezo, J.-L., Prévôt, A.S.H., Baltensperger, U., 2013. Wintertime aerosol chemical composition and source apportionment of the organic fraction in the metropolitan area of Paris. Atmos. Chem. Phys. 13, 961–981. https://doi.org/10.5194/acp-13-961-2013

Elser, M., Huang, R.-J.J., Wolf, R., Slowik, J.G., Wang, Q., Canonaco, F., Li, G., Bozzetti, C., Daellenbach, K.R., Huang, Y., Zhang, R., Li, Z., Cao, J., Baltensperger, U., El-Haddad, I., André, P., Prévôt, A.S.H., 2016. New insights into PM2.5 chemical composition and sources in two major cities in China during extreme haze events using aerosol mass spectrometry. Atmos. Chem. Phys. 16, 3207–3225. https://doi.org/10.5194/acp-16-3207-2016

Katz, E.F., Guo, H., Campuzano-Jost, P., Day, D.A., Brown, W.L., Boedicker, E., Pothier, M., Lunderberg, D.M., Patel, S., Patel, K., Hayes, P.L., Avery, A., Hildebrandt Ruiz, L., Goldstein, A.H., Vance, M.E., Farmer, D.K., Jimenez, J.L., DeCarlo, P.F., 2021. Quantification of cooking organic aerosol in the indoor environment using aerodyne aerosol mass spectrometers. Aerosol Sci. Technol. 55, 1099–1114. https://doi.org/10.1080/02786826.2021.1931013

Xu, W., He, Y., Qiu, Y., Chen, C., Xie, C., Lei, L., Li, Z., Sun, J., Li, J., Fu, P., Wang, Z., Worsnop, D.R., Sun, Y., 2020. Mass spectral characterization of primary emissions and implications in source apportionment of organic aerosol. Atmos. Meas. Tech. 13, 3205–3219. https://doi.org/10.5194/amt-13-3205-2020

Xu, W., Lambe, A., Silva, P., Hu, W., Onasch, T., Williams, L., Croteau, P., Zhang, X., Renbaum-Wolff, L., Fortner, E., Jimenez, J.L., Jayne, J., Worsnop, D., Canagaratna, M., 2018. Laboratory evaluation of species-dependent relative ionization efficiencies in the Aerodyne Aerosol Mass Spectrometer. Aerosol Sci. Technol. 52, 626–641. https://doi.org/10.1080/02786826.2018.1439570
